# Characteristics of teaching auriculotherapy/auricular acupuncture to healthcare professionals worldwide: a scoping review

**DOI:** 10.1590/0034-7167-2024-0512

**Published:** 2025-11-03

**Authors:** Daiana Cristina Wickert, Daniela Dallegrave, Oclaris Lopes Munhoz, Fernanda dos Santos Trombini, Maria Denise Schimith

**Affiliations:** I Universidade Federal de Santa Maria. Santa Maria, Rio Grande do Sul, Brazil; II Universidade Federal do Rio Grande do Sul. Porto Alegre, Rio Grande do Sul, Brazil; III Universidade Federal de Santa Maria. Palmeira das Missões, Rio Grande do Sul, Brazil

**Keywords:** Auriculotherapy, Acupuncture, Ear, Health Personnel, Teaching, Education, Auriculoterapia, Acupuntura Auricular, Personal de Salud, Enseñanza, Educación

## Abstract

**Objectives:**

to map the characteristics of the training of healthcare professionals in auriculotherapy/auricular acupuncture worldwide.

**Methods:**

a scoping review, according to JBI, developed in eight sources of information. Studies that addressed training in auriculotherapy were included, regardless of the design. Descriptive and narrative analysis and synthesis were carried out.

**Results:**

fourteen productions were included, most of which were articles (n=9; 64.3%), available in English (n=10; 71.4%). No course claimed to be fully online, with a workload between 3 and 228 hours. Brazil and France train auriculotherapy practitioners. The United States of America, Australia and Ukraine direct training with an emphasis on specific protocols. Regarding trainers, Brazil and Australia have multidisciplinary teams developing and delivering courses/classes.

**Final Considerations:**

it was identified that training occurs differently around the world, lacking defined minimum requirements. No research was found that assessed training guidelines, research necessary for advancing the provision of care.

## RESUMEN

**Objetivos:**

mapear las características de la formación de profesionales de la salud en auriculoterapia/acupuntura auricular a nivel mundial.

**Métodos:**

revisión del alcance, según el JBI, desarrollada a partir de ocho fuentes de información. Se incluyeron estudios que abordaron la formación en auriculoterapia, independientemente del diseño. Se realizó análisis y síntesis descriptivos y narrativos.

**Resultados:**

se incluyeron 14 producciones, la mayoría de las cuales fueron artículos (n=9; 64,3%), disponibles en inglés (n=10; 71,4%). Ningún curso afirmó ser completamente online, con una carga horaria de entre 3 y 228 horas. Brasil y Francia forman auriculoterapeutas. Estados Unidos de América, Australia y Ucrania dirigen la capacitación con énfasis en protocolos específicos. En cuanto a los formadores, Brasil y Australia cuentan con equipos multidisciplinarios que desarrollan e imparten cursos/clases.

**Consideraciones Finales:**

se identificó que la capacitación se da de manera diferente en todo el mundo, careciendo de requisitos mínimos definidos. No se recuperaron investigaciones que evaluaran pautas de capacitación, investigaciones necesarias para avanzar en la prestación de atención.

## INTRODUCTION

Complementary and alternative medicines (CAM), recognized in the international scientific literature, or also designated by the World Health Organization (WHO) as traditional, complementary and integrative medicines (TCIM), are practices, products and knowledge used by different peoples and cultures around the world. The TCIM care model considers individuals in their singularity and integrality, as well as the sociocultural context in which they live, thus being a model of humanization of care^(1)^. TCIM can be applied by trained therapists and trained healthcare professionals^(2)^.

Within the scope of TCIM, there is auriculotherapy, which in 1990 was recognized by the WHO as a microsystem therapy for promoting and maintaining health in the treatment of various diseases, performed by stimulating points on the external ear^(3)^. There is no consensus on the origin of auricular therapy, which has been used for thousands of years in several countries; however, it can be stated that the greatest development of the technique and the first registered documents are from China^(4)^.

The earliest non-Chinese documentation of ear acupuncture dates back to ancient Egypt, where the treatment of gynecological problems was mentioned in the Ebers Papyrus in 1500 B.C^(5)^. Thus, considering the history of auriculotherapy, when thinking about clinical practice and training professionals, one cannot ignore the three global approaches, such as ear reflexology, auriculotherapy according to traditional Chinese medicine (TCM) and neurophysiology, which coexist with each other with their differences and similarities.

Considering the lack of consensus among auricular maps^(3)^, it can be deduced that the training of professionals who work with auriculotherapy and/or auricular acupuncture also does not have well-defined criteria in different countries around the world. TCM is widely used worldwide, with 170 of the 194 WHO member states recognizing its use. However, in 2018 [most recent information available], only 78 countries indicated that they regulate practitioners. WHO’s support for regulation includes the publication of ten benchmarks for training and seven benchmarks for practice^(6)^.

The WHO benchmarks for the practice of acupuncture, published in 1999 and updated in 2020, is the result of a rigorous process of mapping and reviewing the standards for training specialists in this health approach. The document is divided into: (1) introduction; (2) training categories; (3) training levels; (4) components and requirements for complete training; (5) components and requirements for adapted training. These five parts constitute a complete set of benchmarks for acupuncture training; thus, the document can serve as a basis for establishing training, examination and licensing systems that support qualified practice of acupuncture^(7)^.

However, the document in question does not specifically address training in auriculotherapy and/or auricular acupuncture, and there is no benchmark for this purpose. Therefore, considering that training in auriculotherapy can be provided within specialization training in acupuncture, but also separately, there is a need to know the existing benchmark parameters.

In this regard, this scoping review demonstrates how training in auriculotherapy and/or auricular acupuncture has been occurring around the world, which can provide support for the development of minimum parameters in future research as well as for courses related to the practice.

## OBJECTIVES

To map the characteristics of the training of healthcare professionals in auriculotherapy/auricular acupuncture worldwide.

## METHODS

### Ethical aspects

All ethical principles were respected. The veracity of results was maintained, and the studies were duly cited. However, since this was a review study, assessment by the Research Ethics Committee was waived.

### Study planning and design

A preliminary search was carried out in the PROSPERO, COCHRANE, JBI Evidence Synthesis, PubMed and Open Science Framework (OSF) sources on March 10, 2024, and no review that addressed the problem in question was identified. Thus, the review protocol was registered on the OSF platform, under registration number DOI 10.17605/OSF.IO/5J86R, which can be accessed in full at the link https://osf.io/5j86r/.

This is a scoping review prepared in accordance with JBI methodological stages^(8)^ and reported in accordance with the recommendations of the Preferred Reporting Items for Systematic Reviews and Meta-Analyses Extension for Scoping Reviews (PRISMA-ScR)^(9)^. Nine stages were followed^(8)^: (1) define and align the purpose and question; (2) develop and align inclusion criteria with the purpose and question; (3) describe the planned approach to searching, selecting, extracting and mapping evidence; (4) search for evidence; (5) select evidence; (6) extract evidence; (7) analyze the results; (8) present the results; and (9) summarize evidence in relation to the purpose of the review, draw conclusions and note any implications of conclusions.

### Review question identification

The review question was structured using the mnemonic PCC (Population, Concept and Context), with Population being healthcare professionals, Concept being training/qualification in auriculotherapy or auricular acupuncture, and Context being all contexts in which training takes place. Thus, the following review question was developed: how does the training/qualification of healthcare professionals in auriculotherapy or auricular acupuncture occur, regardless of the context?

### Criteria for selection and identification of relevant studies

Primary studies, experience reports, editorials, opinion articles, reflection articles, theses, dissertations, manuals, guidelines or guidelines, without restrictions on language, time frame or geographic delimitation, were included. Studies conducted simultaneously with undergraduate students and healthcare professionals in which the data could not be separated for analysis were excluded. No language or time frame was defined, and duplicate productions were considered once.

The search strategies were inserted into the specific websites of each source on August 18, 2024, via the Coordination for the Improvement of Higher Education Personnel (In Portuguese, *Coordenação de Aperfeiçoamento de Pessoal de Nível Superior* - CAPES) Portal, through remote access of the *Universidade Federal de Santa Maria* Federated Academic Community (In Portuguese, *Comunidade Acadêmica Federada* - CAFe). Five databases were used, such as EMBASE (Elsevier), Web of Science (WoS), Medical Literature Analysis and Retrieval System Online (MEDLINE), via PubMed, Scopus (Elsevier), and Models of Health and Traditional, Complementary and Integrative Medicines in the Americas (MOSAICO - VHL TCIM), via the Virtual Health Library (VHL). Moreover, searches were carried out in grey literature, such as the *Biblioteca Digital Brasileira de Teses e Dissertações* (BDTD), CAPES Theses and Dissertations Portal, and OpenGrey. The reference list of included studies was also analyzed.

### Study search, screening and selection strategies

Specific search strategies were defined for each data source after a pilot test in MEDLINE and extensive mapping of controlled terms and synonyms in the Medical Subject Headings Section (MeSH), Emtree terms and Health Sciences Descriptors (DeCS) in Spanish, English and Portuguese. The strategies were developed and discussed during the development of a review protocol during a course in a doctoral program. The strategies were extensively tested in the databases, aiming for a highly sensitive search that respected the specificities of each source and retrieved the maximum number of articles that answered the review question. Chart 1 shows the mapping of terms and the test of combining descriptors with appropriate Boolean operators.

**Chart 1:** Search strategies, Santa Maria, Rio Grande do Sul, Brazil, 2024

Database	Search strategies (08/18/2024)
MOSAICO	*auriculoterapia* OR “*acupuntura auricular*” OR “*Acupuntura na Orelha*” OR *auriculoacupuntura* OR *auriculopuntura* OR “acupuntura *en la oreja*” OR “Acupuncture, Ear” OR “auricular acupuncture” OR Auriculot*
MEDLINE	(auriculot* OR “ear acupuncture” OR “auricular acupuncture” OR “acupuncture earlobe” OR auriculoacupuncture) AND (Teaching OR education)
EMBASE	(auriculot* OR auriculotherap* OR ‘ear acupuncture’ OR ‘auricular acupuncture’ OR ‘auriculo-acupuncture’ OR auriculoacupuncture) AND (credentialing OR teaching OR workshop OR education OR ‘training program’)
Scopus	(TITLE-ABS-KEY ( ( auriculot* OR “ear acupuncture” OR “auricular acupuncture” OR “auriculo-acupuncture” OR auriculoacupuncture ) ) AND TITLE-ABS-KEY ( teaching OR workshop OR education OR “Training Program” ) )
Web of Science	(auriculot* OR “ear acupuncture” OR “auricular acupuncture” OR “auriculo-acupuncture” OR auriculoacupuncture OR auriculopuncture) (All Fields) AND “Training Program” OR Teaching OR workshop OR education (All Fields)
*Biblioteca Digital Brasileira de Teses e Dissertações*	*auriculoterapia* OR “*acupuntura* *auricular*” OR “*Acupuntura* *na* *Orelha*” OR *auriculoacupuntura*
CAPES Theses and Dissertations Portal	*auriculoterapia* OR “*acupuntura* *auricular*” OR “*Acupuntura* *na* *Orelha*” OR *auriculoacupuntura*
OpenGrey	auriculot* OR “ear acupuncture” OR “auricular acupuncture” OR “acupuncture earlobe” OR “auriculo-acupuncture” OR auriculoacupuncture

After the searches, the results were exported to the EndNote Web reference manager, where duplicates were excluded. The results were then sent to the Rayyan QCRI application/website for analysis and independent/blind selection, carried out by two PhD reviewers and members of the research group to which the production is linked. After the assessments, in a consensus meeting, divergences were checked, in which the third reviewer participated, but did not need to intervene. It is mentioned that the titles and abstracts were read first (stage 1), and a full reading was carried out in a second selection (stage 2).

### Data extraction

This stage was also conducted by two reviewers. A specific instrument was developed in Microsoft Excel, extracting information on reference, type of document, country, area of knowledge, educational institution, language, population, objective, type of training, duration/workload, program content and modality (online, in-person or blended).

### Data analysis

Data analysis was performed descriptively and narratively, according to the similarity of results, which were organized into categories. Absolute and relative frequencies, figures and charts were also used.

## RESULTS

**Figure 1:**
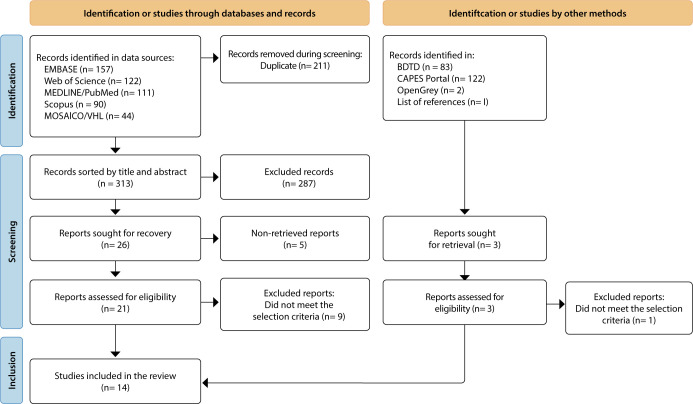
PRISMA ScR flowchart of study search and selection. Santa Maria, Rio Grande do Sul, Brazil, 2024

Based on the search strategies, it was possible to identify 524 productions, of which 211 were duplicates. Thus, in the selection phase by title and abstract, 313 productions were read. Of these, 287 were excluded because they did not meet the selection criteria. In the next stage, 26 records were selected for full reading, of which five were not available (access possibilities were exhausted), being excluded from the review. Therefore, 21 productions were read in full, at which stage nine were excluded. In the searches of grey literature and reference lists, 208 records had their titles and abstracts read, of which three were accessed in full, of which one was excluded because it did not meet the selection criteria. Thus, 14 records constituted the synthesis of evidence (Figure 1).

Chart 2 presents characteristics relating to the population that took the course, the type of training/qualification they received, the workload and teaching method of the auriculotherapy training courses, the institutions involved in training, and their geographical location (country).

**Chart 2:** Characteristics of training described in the studies that make up the sample, Santa Maria, Rio Grande do Sul, Brazil, 2024

Authors (year)	Population that took the course	Type of training/degree	Workload Teaching modality	Institutions involved in offering training and country
Oyola-Santiago; Knopf; Robin; Harvey (2013)^(10)^	No information	Acupuncture detox specialists	- 70 hours - In-person	- The New School in New York - USA
King; Hickey; Connelly (2013)^(11)^	Military healthcare providers	Not specified	- 4 to 8 hours - In-person, with didactic teaching and clinical practice, followed by a short exam	- Air Force Acupuncture Center located at Andrews AFB, Maryland - USA
Stanton; Rangon (2014)^(12)^	Physicians	Inter-University Diploma under the guidance of Dr. David Alimi	- 228 hours of classes (200 hours of theory and 28 practical hours) - In-person, lasting two years, spread over seven weekends, from November to May, annually, generally taught at the *Institut Gustave Roussy*	- Universities of Paris XIII and XI - France
Ferreira (2016)^(13)^	Higher education healthcare professionals	Continuing education	- Course 1: 8 hours; Course 2: 80 hours - Course 1: does not specify the modality; Course 2: blended learning	- UFSC - Brazil
Niemtzow; Baxter; Gallagher; Pock; Calabria; Drake et al. (2018)^(14)^	Healthcare providers	Acupuncture Training Across Clinical Settings to apply BFA	- 4 hours of classes - In-person	- Air Force, Army and Navy - USA
Haywood; Drake; Condie (2019)^(15)^	Resident physicians	Training to apply BFA	- 3 hours - In-person, educational lecture given by a certified instructor followed by a practical demonstration	- Physical Medicine and Rehabilitation Residency Program - USA
Tesser; Moré; Santos; da Silva; Farias; Botelho (2019)^(16)^	Higher education healthcare professionals working in PHC	Blended course	- 75 hours of distance learning (five sequential modules) and 5 hours in person - Blended learning	- UFSC - Brazil
Jan (2020)^(17)^	Physicians	Blended course	- Course 1: 4 hours (with an additional 4 hours online), on the body and BFA at the 35^th^ Scientific Meeting of Emergency Medicine Course 2: 2 hours (with an additional 2 hours, online), held at the test site for emergency room staff who administered only the BFA - Blended learning	- Australasian College for Emergency Medicine - Australia
Jan (2020)^(18)^	Emergency room physicians and nurses	Workshops	- Does not specify the workload - Online and in person	- Emergency Department, St John of God Murdoch Hospital - Australia
Silva; Barros LCN de, Barros NF de; Teixeira; Oliveira (2021)^(19)^	- Course 1: nutritionist and pharmacist - Course 2: pharmacist - Course 3: nurse	Training	- Course 1: 80 hours Course 2: 40 hours Course 3: 12 hours (8 hours of theory and 4 hours of practice) - Course 1: theoretical part online and practical part in person; Course 2: in person; Course 3: in person	- UFSC, Municipal Health Department of Goiânia and Regional Nursing Council of Goiás - Brazil
Hildebrand (2023)^(20)^	Physicians, psychologists, physiotherapists, military paramedics and professionals who identify with some connection to counseling and therapeutic activities	Continuing education	- 14 hours - In person	- Uzhhorod National University College of Medicine - Ukraine
Castle; Lukkahatai; Billing; Huang; Wu; Zhang et al (2023)^(21)^	Oncology nurses	Continuing education (workshop)	- 8 hours (5 teaching hours and 3 practical hours) - In-person	- Comprehensive Cancer Center in Maryland - USA
Hohenberger; Dallegrave (2016)^(22)^	Higher education healthcare professionals working in PHC	Training	-75 hours of distance learning (five sequential modules) and 5 hours of in-person teaching - Blended learning	- UFSC - Brazil
Domingues (2019)^(23)^	Higher education healthcare professionals working in PHC	Training	-75 hours of distance learning (five sequential modules) and 5 hours of in-person teaching - Blended learning	- UFSC - Brazil

*ID - identification; PHC - primary healthcare; BFA - Battlefield Acupuncture; USA - United States of America; UFSC - Universidade Federal de Santa Catarina.*

Among the 14 (100%) productions included, nine (64.3%) were articles^(11,12,14-16,18,19,21,22)^; two (14.4%) were dissertations^(13,23)^; one was a production linked to oral presentation^(17)^ (7.1%); and editorial^(20)^ (7.1%) and brief report^(10)^ (7.1%) represented one document each. As for language, ten (71.4%) were available in English^(10-12,14-18,20,21)^, and four (28.6%), in Portuguese^(13,19,22,23)^. Regarding the year of publication, the documents date from 2013 to 2023, with a predominance in 2019 (n=3; 21.4%)^(15,16,23)^.

Among the training populations, professionals with higher education in Brazilian primary healthcare (PHC)^(13,16,19,22,23)^, graduated from the course offered by the Ministry of Health in partnership with the *Universidade Federal de Santa Catarina* (UFSC), stood out. The training made them auriculotherapy practitioners able to apply the technique in a wide range of situations, especially those common in their work routines in the Brazilian Health System (In Portuguese, *Sistema Único de Saúde* - SUS). However, another very different audience from Brazil seems to be predominant in the USA, which are professionals who work in healthcare services caring for soldiers and war veterans, mainly focused on pain treatment^(11)^, using a protocol of five auricular points called Battlefield Acupuncture (BFA)^(14,15)^. This same training appears in Europe, in the form of continuing education^(20)^, developed with physicians, psychologists and physiotherapists in Ukraine. Furthermore, it is worth noting that, in Australia, the BFA course was aimed at physicians and nurses so that they can work in pain treatment and, consequently, reduce the use of opioids in emergency rooms or first aid clinics^(18,19)^.

A single article from the USA presents a continuing education training through a workshop carried out with nurses for the treatment of cancer pain^(21)^. Another document reports training for the National Acupuncture Detoxification Association (NADA) protocol^(10)^, certifying graduate student as acupuncture detoxification specialists (ADSs), i.e., qualified to apply five auricular points for treatment aimed at drug use.

In relation to the course load, the *Diplôme Interuniversitaire* course, under the guidance of Dr. David Alimi, in France, is the most extensive, with 228 hours divided into two years and offered in person. It is worth noting that this course is also one of those that enables medical professionals to work broadly, not focused on just one specific protocol. Another country with this training characteristic is Brazil, where courses on average have 80 hours, but in the blended learning modality of a course by the Ministry of Health in partnership with UFSC. However, professionals refer to the low course load as a negative point, understanding that 20 hours is not enough time to understand TCM^(19)^.

It is worth noting that, despite the training focused only on the NADA protocol, ADSs completed a course with 70 hours of in-person training. The BFA training had an average of 3 to 8 hours of training, being in-person in the USA and Ukraine, and blended in Australia. The USA differs on how many hours of acupuncture training are clinically necessary to be considered practitioners within their respective scopes^(15)^.

In the auricular point acupressure (APA) training^(21)^, after conducting the course, the authors conducted an assessment to examine nurses’ knowledge based on the APA workshop content. The knowledge test consisted of 43 multiple-choice questions and seven open-ended questions (scores ranged from 0 to 100). The questions addressed the theoretical perspective of auriculotherapy, anatomical terminology of the external ear related to the body, auricular diagnostic procedures for locating auricular points, master points of treatment effects, musculoskeletal and sensory auricular points, and how to stimulate auricular points. The mean score on the post-course knowledge test was 93% (standard deviation [SD] = 6.87), showing that 8 hours of training can be considered adequate for applying APA for cancer-related pain.

In countries whose studies were retrieved for this review (USA, Ukraine and Australia), there is evidence of a pattern of training in specific protocols, such as BFA and NADA, and not “general” training as auriculotherapy practitioners, for use in different situations, as in Brazil^(13,16,19,22,23)^ and France^(12)^, countries where training appeared to be broader.

The blended learning modality was observed only in training courses in Brazil and Australia. No course described claimed to be entirely online. A study^(23)^ that addresses the training offered by UFSC indicates that training is an initiation into auriculotherapy, with an indication of the need to continue training through local situations, such as workshops or study groups.

The following is a general description of the productions, categorized into “Program content of auriculotherapy courses and/or auricular acupuncture courses” and “Characteristics of trainers in auriculotherapy and/or auricular acupuncture courses”. Chart 3 summarizes the information that will later be further explored to answer how auriculotherapy training is occurring around the world.

**Chart 3:** Aspects highlighted in the syllabus of auriculotherapy courses, Santa Maria, Rio Grande do Sul, Brazil, 2024

Description of the program content identified in the study
Teaching the NADA protocol, also known as ACU Detox, which consists of stimulating five auricular points (*Shen Men*, sympathetic nervous system, liver, kidney and lung) for drug use or even mental health^(10)^.
Emphasizes general and specific discussions of clinical patient cases, mainly involving pathology and pathophysiology as well as diagnosis and treatment planning. First year: fundamental biological basis of auriculotherapy (including elements such as embryology, anatomy, genetics and neurophysiology). Second year: clinical applications of auriculotherapy pertaining to all areas of medicine (including neurology, cardiology, endocrinology, pulmonary medicine, pediatrics, among others)^(12)^.
Review the history of acupuncture, clinical significance, and evidence for its use; understand patient selection criteria; provide educational information to patients about acupuncture; obtain and document consent; gain proficiency in needling technique (e.g., needle location and placement); identify, understand, and communicate post-procedure instructions and precautions; obtain appropriate credentialing. Lectures, videos, demonstrations and practice with silicone ears and live models were used. Trainees’ learning and proficiency of skills are assessed through trainer observation and written examination. Each training course ends with a written examination. A course assessment is completed at the conclusion of the training^(14)^.
The didactic session focused on the BFA protocol specifics, including safety, proper needle technique, patient selection, and needle care. During the hands-on demonstration, each participant verified their knowledge of the correct placement of five BFA needles according to the protocol. This demonstration was practiced first with silicone ears followed by needle placement in the remaining resident participants^(15)^.
The modules included the following items: (1) introduction to integrative practices; (2) auricular reflexology; (3) introduction to TCM; (4) biomedical view of auriculotherapy; and (5) auriculotherapy in PHC. The teaching material included a handout for each module, 14 video classes and an interactive ear (online resource) for studying the location and application of the main auricular points. The in-person lectures follow a structured script of auricular palpation techniques, practice of inserting auricular seeds and discussion of clinical cases, under the supervision of trained instructors^(16)^.
Content included: evidence, indications for pain, contraindications, application, safety, mechanism of action, and how to negotiate accreditation barriers. The workshops used teaching methods such as problem-based learning, infotainment, simulation, “Peyton’s four-stage skills teaching” and “teaching in motion”. Pre-course learning was primarily provided through videos on: “Overview of Emergency Department (ED) BFA Application, Appropriateness, and Case Selection”; “Current Evidence Supporting ED Auricular Acupuncture”; “How to Perform BFA”; “Mechanisms of Auricular Acupuncture”; and “BFA Safety.” Included in the pre-course package were videos by Dr. Richard Niemtzow on “Opening the Package, Removing the Applicator, and Demonstrating Needle Size” and “Applying BFA Points in Sequence on an Ear,” applying BFA and DuoDERM Tapes, and safely removing needles in the ED setting. The assigned pre-readings included systematic review articles. Simulation on silicone ear models helped teach the dexterity required in using *aiguille semi-permanente* (ASP—Sedatelec, Irigny, France)^(18)^.
Course 1: not specified. Course 2: the course includes the assistance of 24 users, with a report of the assistance and sending it to the course tutor. Course 3: the course has an instructor. The theoretical part covered the origin, functioning, performance and purpose. In practice, the application was carried out with the course colleagues^(19)^.
Auricular trauma protocol, with five needles; Auricular trauma protocol, with six needles; BFA, with ASP needles^(20)^.
The didactic training comprised content from textbooks, articles, and websites, presented in five lectures: (1) overview and theoretical perspective of auriculotherapy; (2) anatomical terminology of the external ear; (3) auricular master points, musculoskeletal, and sensory auricular points; (4) auricular diagnostic and treatment procedures; and (5) commonly used APA protocols for cancer-related pain. For the hands-on training, learning was reinforced through immersive learning, demonstration, and feedback. Nurses practiced identifying appropriate auricular points by placing a pin in silicone earlobes. They then practiced using a locator to identify auricular points in a selected area of cancer-related pain and placing APA seeds on a volunteer nurse while being observed and guided by the instructor. To successfully complete the hands-on course, nurses had to demonstrate competency in APA through a skills checklist, including using a locator to find pain points on the body, placing seeds, and stimulating auricular points^(21)^.
During the distance learning stage, which totaled 75 hours, the five modules gave an idea of what auriculotherapy is, how to perform a diagnosis, possible approaches and how it could be incorporated into the work routine, providing some examples and encouraging people to think about the practice and the work process with auriculotherapy^(22)^.
The first stage is classified as self-instructional, while the second is called preceptorship, which enables the implementation of what was theoretically developed in the modules. The first module begins with the historical process of the presence of ICPs in the SUS and a brief introduction to auriculotherapy. The following three modules cover three approaches to this technique: reflexology, TCM and biomedicine. Each module is dedicated to one of these therapeutic rationales, organizing how to conduct the diagnosis and define treatment. The last module is dedicated to systematizing how this practice can be introduced into SUS services^(23)^.

*PHC - primary healthcare; TCM - traditional Chinese medicine; BFA - Battlefield Acupuncture; NADA - National Acupuncture Detoxification Association; ICPs - integrative and complementary practices; SUS - Brazilian Health System; APA - auricular point acupressure; ED – emergency department.*

### Program content of auriculotherapy and/or auricular acupuncture courses

Three retrieved studies do not present a description of the program content^(11,13,17)^, while the others address aspects of auriculotherapy training, such as biological basis, history, clinical practice, treatment protocols and teaching methods. Among the teaching methods, evidence-based teaching^(14,18)^, simulation and practical application stood out, to ensure the necessary skills in auriculotherapy, as can be seen in Chart 3.

In general, the courses cover theoretical and practical elements, with a focus on safety, proper technique, and correct identification of auricular points. The only training that describes TCM teaching is Brazilian^(16)^, as it presents an introduction to the medical and biomedical explanations of TCM, including neurophysiology and scientific research. The authors justify this choice for two reasons: (1) didactic: based on the experience of in-person teaching, the online content (reflexology – TCM – biomedicine) would be easy to understand; (2) epistemological: by contextualizing the material in each of the three approaches, it avoided reducing or explaining one approach based on the others – usually biomedicine. Thus, the course uses the terms of TCM and auricular reflexology in its approach, not applying the typical methodological procedures used in biomedicine.

According to the contents described in the documents^(12,14,18)^, training in auriculotherapy occurs through a curriculum that covers the fundamental biological basis, clinical applications, theoretical and practical teaching, specific protocols, and evidence-based teaching methods. The training programs include theoretical classes, practical applications, simulations, and discussion of clinical cases. Regarding teaching methods, it is noted that training in the BFA protocol uses silicone ears^(14,15,18,21)^ to handle the needles before training in people. We highlighted a study^(18)^ in which teaching methods such as problem-based learning, infotainment, simulation, “Peyton’s four-stage skills teaching” and “teaching in motion” are described.

The Grundy Confidence Scale (1993)^(24)^ was administered to oncology nurses before and after training, using a self-report measure consisting of one item adapted from the scale: overall, how confident are you that you are able to successfully practice APA? The item was measured using a 10-point Likert scale, ranging from 0 (not at all confident) to 10 (extremely confident). Thus, the mean score on the confidence measure in APA practice increased from 0 before the workshop to 6.5 (SD = 0.84, range 5 to 10) after the workshop^(21)^.

### Characteristics of trainers in auriculotherapy and/or auricular acupuncture courses

Not all of the productions that comprised the *corpus* of this scope detail who were the people who conducted the training or designed the course content. Thus, a study^(15)^ had a BFA instructor who was trained by Dr. Niemtzow at the Air Force Acupuncture Center. In the study^(16)^, a multidisciplinary team created the auriculotherapy course content. Most of team members had previous or current experience as conventional PHC professionals (three physicians, two nurses, one pharmacist and one physiotherapist). All were acupuncturists with experience in the use and/or teaching of auriculotherapy/acupuncture. This team was responsible for preparing all of the course material and formed the course management committee.

The course instructors^(18)^ were emergency practitioners with formal qualifications in general acupuncture, general practitioners (family and community medicine) from Australian Medical Acupuncture College examination faculty and TCM practitioners. The emergency practitioners understood the case mix and which analgesia problems could be addressed. In addition, they knew how to adapt auricular acupuncture techniques to the emergency room environment, while understanding participants’ mindset. General practitioners (family and community medicine) and TCM practitioners contributed to the accurate selection of points, location, and needling techniques. The TCM faculty had excellent knowledge of Chinese cosmological theories, but had reservations about the biomedical model, which was noted in the formal feedback.

Celia Hildebrand, after a 2017 lecture at Uzhhorod National University College of Medicine on the use of auricular acupuncture for pain and addiction, received a Fulbright grant to develop an auricular acupuncture curriculum in Ukraine. Training was organized by Uzhhorod National University College of Medicine and lasted 14 hours, resulting in certification and Continuing Medical Education credits. The curriculum was subsequently refined and delivered again in August 2022, and revised again and delivered in May 2023. In June 2023, the course was taught for the first time in Lviv, Ukraine^(20)^.

## DISCUSSION

Some CAMs have been used for thousands of years. Therefore, it is worth considering that registration and documentation regarding their use may be increasing worldwide. In addition, institutions concerned with training, certification and information are also growing. For instance, in the USA there is the National Center for Complementary and Alternative Medicine, which conducts and supports research and provides information on complementary health products and practices^(25)^. In terms of training, countries are encouraged to assess and recognize WHO benchmarks as minimum standards, as relevant to their national context^(7)^.

Twelve years ago, in October 2012, the First International Congress for Educators in Complementary and Integrative Medicine took place in the USA capital, with the aim of advancing the field of education in complementary and integrative medicine through the sharing of best practices in the development, implementation, assessment and dissemination of curricula and teaching methods^(26)^. The congress can be seen as a milestone in terms of training in these practices.

The WHO Traditional Medicine Strategy 2013-2023 (extended to 2025) is a set of guidelines that aims to support member states in implementing policies and action plans that strengthen the importance of traditional medicine. Among the challenges cited in it is the education and training of traditional medicine practitioners^(2)^.

Turning attention to teaching and training in auriculotherapy, online training courses have been developed, such as the course by Dr. Terry Oleson, a specialist and world leader in auriculotherapy, who provides certification through the Auriculotherapy Certification Institute (ACI)^(27)^. The ACI is an institute created in 1999 to offer training and certification in auriculotherapy in the USA, without exempting practitioners from complying with state regulations. The ACI offers three types of auriculotherapy certification: transcutaneous stimulation; auricular acupuncture with needle insertion; and ear reflexology with tactile manipulation^(27)^. Yet another course of international relevance is by Dr. Raphaël Nogier, physician and researcher^(28)^.

The existence of renowned courses and few experiences of teaching auriculotherapy and/or auricular acupuncture recorded in literature^(12)^ ends up directing the discussion towards examples of acupuncture, a practice that seems to have more defined training standards. Thus, in order to problematize some issues that should be taken into consideration in auriculotherapy training, according to the world literature, some gaps and possibilities will be pointed out.

In Australia, a national registration and accreditation scheme for health professions has been in place since July 2012, incorporating the registration of acupuncturists with the Chinese Medicine Board of Australia. A literature review identified studies on acupuncture education standards, as well as a survey of standards-setting bodies and course providers in the country, but concluded that there is little literature on acupuncture education standards in Australia, despite its practice by a wide range of healthcare professionals^(29)^. Furthermore, in the country, Australian healthcare professionals who practice complementary medicine have been supervised by the Australian Health Practitioner Regulation Agency since 2010, including acupuncturists. Research finds that the qualifications of professionals, educational standards and scope of procedures related to complementary medicine are managed through legal regulations by the federal and state governments^(30)^. No evidence was found regarding standards or records for the practice of auriculotherapy specifically in the country.

A Brazilian study^(19)^ concludes that the training processes in Integrative and Complementary Health Practices (ICHP) (the nomenclature of TCIM or CAM in Brazil) are heterogeneous, deficient and limited. Furthermore, they demonstrate the lack of organization of human resources training and development of qualification strategies in ICHP. A study carried out in Florianópolis, Santa Catarina, Brazil, presents relevant data regarding the lack of use of diagnostic resources and anamnesis of this traditional TCM knowledge before the application of auriculotherapy. The researcher observed that in such practices the diagnosis is performed following standard biomedical logic and then the intervention in ICHP^(13)^. Thus, this aspect reinforces the need for a guideline directing and qualifying training in auriculotherapy according to the bases that originated the practice. Data supports the curricular content identified in the present review, in which only UFSC courses reported the study of TCM.

Considering the contributions that ICHP (including auriculotherapy) can make to comprehensive healthcare, especially positive experiences in Brazilian PHC, a review study states that “[...] there is little investment in its teaching, and it is poorly understood by undergraduate students and professors in health courses. Therefore, it is urgent that ICHP be considered for inclusion in the health education curriculum [...]”^(31)^.

A study conducted in Brazil with 24 managers of Basic Health Units in the Metropolitan Region of Goiânia, Goiás, most of whom were nurses, refers to the lack of in-service training of professionals as an educational barrier to the implementation of ICHP in PHC. Managers state the lack of continuing education on ICHP as a public offering, and it is up to professionals to seek training. In addition, they mention the in-person auriculotherapy course offered by the Goiânia Health Department free of charge. Training in ICHP is one of the critical points for the effective expansion of these practices^(32)^.

The Defense Veteran’s Center for Integrative Pain Management’s important initiative is the education and training of multidisciplinary providers in integrative pain management strategies, highlighting the importance of training as well as funding to support it. The same article developed an auricular acupuncture curriculum to train more than 2,700 professionals from The Department of Defense and Veterans Affairs with responsibilities in pain management since 2016^(14)^. For this pain treatment, several authors use BFA, developed in 2001 by Richard C. Niemtzow, a radiation oncologist for the US Air Force, who adapted the auricular acupuncture technique to be used as a method of rapid pain relief on the battlefield. This is a technique that uses semi-permanent needles that are placed in auricular acupuncture points and can remain in the ear for three to four days^(33)^.

Although BFA is based on ancient TCM, it can be considered a new modality in the midst of a pharmaceutical and interventionist medical community^(34)^. This modality offers a new paradigm, with a practical skill that is outside the current influences of the industry and the pharmaceutical sector. The authors conclude that it can be easily incorporated into a medical residency curriculum, especially those with training that revolves around quality of life, pain control and patient function^(15)^.

When talking about BFA for pain relief in emergency room care, nurses are concerned about the excessive prescription of opioids, and understand that they are the ideal professional category to learn auriculotherapy for use in these cases, due to their proximity to patients and their skills in using BFA, which can be applied in triage^(18)^.

### Study limitations

While the main method and reporting guidelines for scoping review were followed, the difficulty in accessing some productions for analysis in full, resulting in exclusion, constitutes a limitation. Furthermore, the lack of standardization of information in the productions on training in auriculotherapy/auricular acupuncture made other comparisons and inferences difficult.

### Contributions to health, nursing, or public policy

Evidence points to a lack of research that guides, regulates or at least directs the training of healthcare professionals in auriculotherapy/auricular acupuncture. Thus, by bringing into the discussion other practices with more delimited training, such as acupuncture, the scientific evidence of this scope contributes to directing and providing subsidies for the construction of minimum training standards, according to each reality and profession. Therefore, by bringing characteristics of the training of healthcare professionals in auriculotherapy/auricular acupuncture worldwide, this review can serve as a basis for developing, qualifying, deepening, assessing and monitoring guidelines for the training of healthcare professionals in auriculotherapy/auricular acupuncture.

## FINAL CONSIDERATIONS

It was possible to map the characteristics of the training of healthcare professionals in auriculotherapy/auricular acupuncture worldwide. In short, it was found that training in auriculotherapy occurs differently around the world. The convergences are between Brazil and France, which train auriculotherapy practitioners. In other countries, such as the USA, Australia and Ukraine, training is directed towards the use and application of specific protocols, such as BFA and NADA. In addition, the blended modality was observed only in training in Brazil and Australia. No course described claimed to be entirely online, and the workload varied from 228 to 3 hours of teaching. The only training that describes teaching in TCM is Brazilian. Regarding the trainers, two studies had a multidisciplinary team developing and delivering the training content, one of them in Brazil and the other in Australia.

No studies assessing training guidelines for auriculotherapy/auricular acupuncture were retrieved. Therefore, future research along these lines is suggested to advance the provision of care to the population based on qualified training, addressing one of the challenges of the WHO Traditional Medicine Strategy 2013-2023 (extended until 2025). Furthermore, it is necessary to advance research that develops references to establish training, examination and licensing systems that support the qualified practice of auriculotherapy/auricular acupuncture worldwide, respecting each context and profession, in order to arrive at minimum parameters and specific legislation for training/teaching in auriculotherapy/auricular acupuncture.

## Data Availability

The research data are available in a repository: https://osf.io/5j86r/.
