# Supplementary material for: Characteristics of teaching auriculotherapy/auricular acupuncture to healthcare professionals worldwide: a scoping review
Source: Rev Bras Enferm. 2025 Nov 3;78(4):e20240512. doi: 10.1590/0034-7167-2024-0512 (PMC12584949; doi:10.1590/0034-7167-2024-0512)
Supplement: Supplementary file 1 [file 0034-7167-reben-78-04-e20240512-suppl01.pdf]

UNIVERSIDADE FEDERAL DE SANTA MARIA
CENTRO DE CIÊNCIAS DA SAÚDE
PROGRAMA DE PÓS-GRADUAÇÃO EM ENFERMAGEM
CURSO DE DOUTORADO

FORMAÇÃO EM AURICULOTERAPIA OU ACUPUNTURA AURICULAR: protocolo de revisão de escopo

Autora: Daiana Cristina Wickert

Orientadora: Maria Denise Schimith

Coorientadora: Daniela Dallegrave

PROBLEMA DE PESQUISA

A auriculoterapia é uma técnica milenar, que apesar de ser amplamente pesquisada e utilizada para as mais diversas questões, teve uma resolução com maior ênfase na formação na enfermagem em 2024, com a resolução COFEN nº 739 de 05 fevereiro, apresentando, pela primeira vez, uma definição mínima de carga horária de 80 horas para formação em auriculoterapia, como capacitação por meio de cursos livres (CONSELHO FEDERAL DE ENFERMAGEM, 2024). No entanto, ainda carece de critérios mínimos definidos acerca de como se dará essa formação de enfermeiros(as) no Brasil. Apesar de artigos abordarem o panorama de formação na graduação em enfermagem brasileira, recente pesquisa evidenciou que a formação em Práticas Integrativas e Complementares (PICS) dos enfermeiros(as) brasileiros(as) ocorre majoritariamente após a graduação. As evidências apontam para a insuficiência da formação dos profissionais, mostrando um déficit na oferta e qualidade das mesmas.

Na presente revisão, considera-se a formação como algo que objetiva dotar as pessoas de conhecimentos teóricos e práticos, capacidades e/ou competências exigidas por profissões específicas ou pelo mercado de trabalho (EUROPEAN CENTRE OF DEVELOPMENTE OF VOCATIONAL TRAINEING, 2014), sendo está definição adaptada da European Training Foundation de 1997. Assim, entende-se que para a utilização da auriculoterapia por enfermeiros(as), é necessária uma formação após a graduação, que seja participativa e possa construir conhecimentos próprios desta prática milenar específica, aliada aos conhecimentos, saberes e práticas da profissão.

Assim, o problema de pesquisa de revisão é o desconhecimento das evidências acerca dos critérios/diretrizes mínimas para formação em auriculoterapia nos diferentes contextos. Sendo assim, a presente revisão de escopo visa conhecer como a formação em auriculoterapia está ocorrendo no mundo, a fim de buscar subsídios para rever esta realidade na enfermagem brasileira.

Para isso, foi realizada a seguinte busca preliminar no dia 10 de março de 2024, com a estratégia (auriculotherapy OR acupuncture, ear) AND (Credentialing OR Education OR Teaching) na PROSPERO (26 registros), OSF (a busca foi realizada apenas com "auriculotherapy") (7 resultados), COCHRANE (53 trials e 0 reviews), JBI Evidence Synthesis (1) e PUBMED (114).

Sendo assim, constatou-se que não existe pesquisa de revisão que responda ao problema em questão. Nesse sentido, desenvolver essa revisão irá auxiliar a compreender melhor como a formação em auriculoterapia ocorre no mundo, a fim de guiar a pesquisa de campo que será desenvolvida na tese e que tem por objetivo "analisar a formação de enfermeiros(as) em auriculoterapia no Brasil e construir uma proposta de diretriz para formação qualificada".

Destaca-se que não foi delimitado apenas a formação de enfermeiros, devido ao baixo número de estudos recuperados. Na busca preliminar realizada, foram encontrados três artigos que respondem à questão de revisão, estando os mesmos indicados abaixo:

Hohenberger GF, Dallegrave D. Auriculoterapia para profissionais de saúde: percursos possíveis da aprendizagem à implantação na unidade de saúde. Saúde em Redes. 2016;2(4):372–82. Available from: http://revista.redeunida.org.br/ojs/index.php/rede-unida/article/view/802.

Tesser CD, Moré AOO, Santos MC, da Silva EDC, Farias FTP, Botelho LJ. Auriculotherapy in primary health care: A large-scale educational experience in Brazil. J Integr Med. 2019 Jul;17(4):302-309. doi: 10.1016/j.joim.2019.03.007.

Silva, P. H. B. da ., Barros, L. C. N. de ., Barros, N. F. de ., Teixeira, R. A. G., & Oliveira, E. S. F. de .. (2021). Formação profissional em Práticas Integrativas e Complementares: o sentido atribuído por trabalhadores da Atenção Primária à Saúde. Ciência & Saúde Coletiva, 26(2), 399–408. https://doi.org/10.1590/1413-81232021262.40732020.

Assim, o presente protocolo de revisão de escopo possui a questão de revisão estruturada pelo mnemônico P (população) C (conceito) C (contexto): COMO OCORRE A FORMAÇÃO/QUALIFICAÇÃO DE PROFISSIONAIS DA SAÚDE EM AURICULOTERAPIA E/OU ACUPUNTURA AURICULAR, INDEPENDENTEMENTE DO CONTEXTO?

Os critérios de inclusão dos estudos primários para cada um dos elementos do acrônimo estão descritos no quadro abaixo:

Acrônimo	Elemento da questão	Critério de inclusão
P – População	Profissionais da saúde	Estudos com qualquer profissional da saúde que possua alguma formação em auriculoterapia ou que relate o processo de formação dos mesmos
C – Conceito	Formação em auriculoterapia	Estudos que abordem qualquer tipo de formação em auriculoterapia ou acupuntura auricular, seja aperfeiçoamento, qualificação profissional,

		minicurso, curso de extensão, treinamento, pós-graduação, formação informal ou autodeclarada.
C – Contexto	Em qualquer contexto	Realizados em qualquer contexto (atenção primária à saúde, hospital, domicílio, centro de atenção psicossocial, policlínica, clínica particular)

Tipo de estudos: estudos primários, relatos de experiência, editoriais, artigos de opinião, artigos de reflexão, teses, dissertações, manuais, diretrizes ou *guidelines*.

Idioma: não será delimitado.

Delimitação geográfica: não será estabelecida.

Recorte temporal: não será estabelecido.

Já os critérios de exclusão serão estudos que abordem estudantes de graduação e estudos realizados com estudantes de graduação e profissionais da saúde e que os dados não possam ser separados para análise.

As bases de dados utilizadas serão:

-MOSAICO via BVS MTCI- Modelos de saúde e Medicinas Tradicionais, Complementares, e Integrativas nas Américas (por ser uma base de dados das MTCI)

-EMBASE (devido sua representatividade na Europa)

-Medline via portal PUBMED (devido sua abrangência internacional)

-SCOPUS (por se tratar de uma base de dados interdisciplinar)

-Web Of Science (por se tratar de uma base de dados de importância internacional)

-Será realizada buscas na literatura cinzenta: Biblioteca Digital Brasileira de Teses e Dissertações, Portal de Teses e dissertações da CAPES, Google Scholar e OpenGray. Ainda, será avaliada a lista de referências dos estudos incluídos.

O mapeamento dos termos que foram compilados estão descritos no quadro abaixo, separados de acordo com o mnemônico PCC:

**Elementos do acrônimo/ Mnemônico**	**DECS**				**MESH/Entry Terms**			
	Descritor	Sinônimos	Definição	Ano de indexação	Descritor	Sinônimos	Definição	Ano de indexação
População<br>Profissionais da saúde	**Português:**<br>Pessoal de Saúde<br><br>**Espanhol:**<br>Personal de Salud<br><br>**Francês:**<br>Personnel de santé	Pessoal da Saúde<br>Prestadores de Cuidados de Saúde<br>Profissionais da Saúde<br>Profissionais de Saúde<br>Profissional da Saúde<br>Profissional de Saúde<br>Trabalhador da Saúde<br>Trabalhador de Saúde<br>Trabalhadores da Saúde<br>Trabalhadores de Saúde<br><br>Profesionales de la Salud<br>Proveedores de Atención de Salud<br>Trabajadores de la Salud<br><br>Personnel sanitaire<br>Prestataires de soins<br>Prestataires de soins de santé<br>Professionnels de la santé	Indivíduos que trabalham na provisão de serviços de saúde, quer como médicos individuais ou empregados de instituições e programas de saúde, profissionais de saúde treinados ou não, sujeitos ou não a regulamento público.	1999	Health personnel	Personnel, Health<br>Health Care Providers<br>Health Care Provider<br>Provider, Health Care<br>Healthcare Providers<br>Healthcare Provider<br>Provider, Healthcare<br>Healthcare Workers<br>Healthcare Worker<br>Health Care Professionals<br>Health Care Professional<br>Professional, Health Care	Men and women working in the provision of health services, whether as individual practitioners or employees of health institutions and programs, whether or not professionally trained, and whether or not subject to public regulation. (From A Discursive Dictionary of Health Care, 1976)	1992
Conceito<br>Formação em auriculoterapia	**Português:**<br>auriculoterapia<br><br>**Espanhol:**<br>Auriculoterapia<br><br>**Francês:**<br>Auriculothérapie	-<br><br>-<br><br>-	Tratamento de dor, vícios em drogas ou outros males pela estimulação de vários pontos na orelha externa (PAVILHÃO AURICULAR). É baseada em	2008	Auriculotherapy	Auriculotherapies	Treatment of pain, drug addictions, or other ailments by stimulating the various points on the external ear (EAR AURICLES). It is based on the ancient Chinese	2009

			práticas chinesas ancestrais de ACUPUNTURA AURICULAR, mas algumas vezes são utilizados ímãs e outras formas de estímulo.				practices of EAR ACUPUNCTURE, but sometimes magnets and other modes of stimulation are used.	
	**Português:** Acupuntura Auricular<br><br>**Espanhol:** Acupuntura Auricular<br><br>**Francês:** Acupuncture auriculaire	Acupuntura na Orelha<br>Auriculoacupuntura<br>Auriculopuntura<br><br>acupuntura en la oreja	Terapia de acupuntura por inserção de agulhas na orelha. É utilizada para controle da dor e tratamento de várias enfermidades.	1999	**MESH:** Acupuncture, Ear<br><br>**EMTREE:** auricular acupuncture	Acupunctures, Ear<br>Ear Acupunctures<br>Auricular Acupuncture<br>Ear Acupuncture<br>Acupuncture, Auricular<br>Acupunctures, Auricular<br>Auricular Acupunctures<br><br>acupuncture, earlobe<br>auriculo-acupuncture<br>auriculoacupuncture	Acupuncture therapy by inserting needles in the ear. It is used to control pain and for treating various ailments.	2000
	**Português:** Credenciamento<br><br>**Espanhol:** Habilitación Profesional<br><br>**Francês:** Délivrance de titres et certificats	Habilitação Profissional<br>Qualificação Profissional<br>Qualificação Profissional em Saúde<br><br>diplomatura<br>licenciatura	Reconhecimento da competência técnica ou profissional através de registros, certificados, licenciamento, admissão em sociedades ou associações, diplomas etc.	1978	Credentialing	-	The recognition of professional or technical competence through registration, certification, licensure, admission to association membership, the award of a diploma or degree, etc.	1980

	**Português:**<br>capacitação profissional<br><br>**Espanhol:**<br>Capacitación Profesional<br><br>**Francês:**<br>Formation Professionnelle	Formação Profissional<br><br>formación profesional	Treinar uma pessoa ou um grupo de pessoas no conhecimento ou na aplicação prática e teórica de uma determinada atividade.	-	-	-	-	-
	**Português:**<br>Ensino	Atividade de Treinamento<br>Atividades Formativas<br>Atividades de Capacitação<br>Atividades de Formação<br>Atividades de Treinamento<br>Atividades de Treino<br>Capacitação Acadêmica<br>Didática<br>Docência<br>Formação Acadêmica<br>Método de Ensino<br>Métodos Pedagógicos<br>Métodos de Ensino<br>Pedagogia<br>Treinamento Acadêmica<br>Treino Acadêmico<br>Técnica de Treinamento<br>Técnicas Educacionais<br>Técnicas Educativas<br>Técnicas de Ensino<br>Técnicas de Formação<br>Técnicas de Treinamento<br>Técnicas de Treino	Processo formal e organizado de transmissão de conhecimento para uma pessoa ou grupo.	1999	Teaching	Training Techniques<br>Training Technique<br>Technique, Training<br>Techniques, Training<br>Training Technics<br>Technic, Training<br>Technics, Training<br>Training Technic<br>Pedagogy<br>Pedagogies<br>Teaching Methods<br>Teaching Method<br>Method, Teaching<br>Methods, Teaching<br>Academic Training<br>Training, Academic<br>Training Activities<br>Training Activity<br>Activities, Training<br>Activity, Training<br>Techniques, Educational<br>Educational Techniques<br>Educational Technique<br>Technique, Educational<br>Educational Technics	A formal and organized process of transmitting knowledge to a person or group.	-

	**Espanhol:**<br>Enseñanza<br><br>**Francês:**<br>Enseignement	actividad formativa<br>docencia<br>método de enseñanza<br>pedagogía<br>técnica formativa				Educational Technic<br>Technic, Educational<br>Technics, Educational<br>education servisse<br>educational measurement<br>intellectual training<br>training support.		
	**Português:**<br>Educação<br><br>**Espanhol:**<br>Educación<br><br>**Francês:**<br>Éducation	Atividades Educacionais<br>Atividades Educativas<br>Atividades Socioeducativas<br>Educar<br>Oficinas de Trabalho<br>Programas de Alfabetização<br>Programas de Treinamento<br>Workshops<br><br>actividades educativas<br>programas de alfabetización<br>programas de formación<br>talleres	Aquisição de conhecimento como resultado de instrução em um curso formal de estudo.	1999	Education	Workshops<br>Workshop<br>Training Programs<br>Program, Training<br>Programs, Training<br>Training Program<br>Educational Activities<br>Activities, Educational<br>Activity, Educational<br>Educational Activity<br>Literacy Programs<br>Literacy Program<br>Program, Literacy<br>Programs, Literacy	Acquisition of knowledge as a result of instruction in a formal course of study.	-
	**Português:**<br>Capacitação em Serviço<br><br>**Espanhol:**<br>Capacitación en Servicio	Programas de Orientação ao Empregado<br>Treinamento em Serviço<br><br>capacitación del personal<br>entrenamiento en servicio<br>programas de orientación a los empleados	Sobre os programas de capacitação no trabalho para o pessoal realizar dentro de uma instituição ou agência. Inclui	1999	Inservice Training	On-the-Job Training<br>On the Job Training<br>Training, On-the-Job<br>Training, Inservice<br>Orientation Programs, Employee<br>Employee Orientation Program	On the job training programs for personnel carried out within an institution or agency. It includes orientation programs.	1970

	**Francês:** Formation en interne		programas de orientação.			Orientation Program, Employee Program, Employee Orientation Programs, Employee Orientation Employee Orientation Programs		
Contexto Em qualquer contexto	-	-	-	-	-	-	-	-

Teste de combinação dos descritores com os operadores adequados (OR, AND, AND NOT) para MOSAICO:

CONTROLE DE ESTRATÉGIAS REALIZADAS 18/08/2024					
**FONTES**	**ACRÔNIMO**	**ELEMENTO DA PERGUNTA**	**#**	**ESTRATÉGIA**	**TOTAL RECUPERADO**
MOSAICO/ Biblioteca Virtual em Saúde	P	Profissionais da saúde	#1	"Personnel, Health" OR "Health Care Providers" OR "Health Care Provider" OR "Provider, Health Care" OR "Healthcare Providers" OR "Healthcare Provider" OR "Provider, Healthcare" OR "Healthcare Workers" OR "Healthcare Worker" OR "Health Care Professionals" OR "Health Care Professional" OR "Professional, Health Care" OR "Health personnel" OR "Pessoal de Saúde" OR "Pessoal da Saúde" OR "Prestadores de Cuidados de Saúde" OR "Profissionais da Saúde" OR "Profissionais de Saúde" OR "Profissional da Saúde" OR "Profissional de Saúde" OR "Trabalhador da Saúde" OR "Trabalhador de Saúde" OR "Trabalhadores da Saúde" OR "Trabalhadores de Saúde" OR "Profesionales de la Salud" OR "Proveedores de Atención de Salud" OR "Trabajadores de la Salud" OR "Personnel sanitaire" OR "Prestataires de soins" OR "Prestataires de soins de santé" OR "Professionnels de la santé"	102
	C	Auriculoterapia	#2	auriculoterapia OR "acupuntura auricular" OR "Acupuntura na Orelha" OR auriculoacupuntura OR auriculopuntura OR "acupuntura en la oreja" OR "Acupuncture, Ear" OR "auricular acupuncture" OR Auriculot*	44
	C	Formação	#3	Credentialing OR Workshop OR "Training Program" OR "Educational Activities" OR "Activity, Educational" OR Teaching OR "Inservice Training" OR "educational measurement" OR "intellectual training" OR "training support" OR credenciamento OR “capacitação profissional” OR “capacitación profesional” OR “formation professionnelle”	0

				OR ensino OR enseñanza OR enseignement OR educação OR educación OR éducation OR “capacitación en servicio” OR “qualificação profissional” OR diplomatura OR licenciatura OR “formação profissional” OR “formación profesional" OR didática OR “técnicas educacionais” OR “actividad formativa” OR docência OR pedagogia OR educar OR “programas de treinamento” OR workshops OR “programas de alfabetización” OR “programas de formación” OR talleres OR “capacitación del personal”	
	C	Em qualquer contexto		-	-
	**Combinando estratégias**				
	#3#2	Credentialing OR Workshop OR "Training Program" OR "Educational Activities" OR "Activity, Educational" OR Teaching OR "Inservice Training" OR "educational measurement" OR "intellectual training" OR "training support" OR credenciamento OR “capacitação profissional” OR “capacitación profesional” OR “formation professionnelle” OR ensino OR enseñanza OR enseignement OR educação OR educación OR éducation OR “capacitación en servicio” OR “qualificação profissional” OR diplomatura OR licenciatura OR “formação profissional” OR “formación profesional" OR didática OR “técnicas educacionais” OR “actividad formativa” OR docência OR pedagogia OR educar OR “programas de treinamento” OR workshops OR “programas de alfabetización” OR “programas de formación” OR talleres OR “capacitación del personal” AND auriculoterapia OR "acupuntura auricular" OR "Acupuntura na Orelha" OR auriculoacupuntura OR auriculopuntura OR "acupuntura en la oreja" OR "Acupuncture, Ear" OR "auricular acupuncture" OR Auriculot*			0
	**Estratégia escolhida**				
	#2	auriculoterapia OR "acupuntura auricular" OR "Acupuntura na Orelha" OR auriculoacupuntura OR auriculopuntura OR "acupuntura en la oreja" OR "Acupuncture, Ear" OR "auricular acupuncture" OR Auriculot*			44

Teste de combinação dos descritores com os operadores adequados (OR, AND, AND NOT) para MEDLINE:

**CONTROLE DE ESTRATÉGIAS REALIZADAS 18/08/2024**					
**FONTES**	**ACRÔNIMO**	**ELEMENTO DA PERGUNTA**	**#**	**ESTRATÉGIA**	**TOTAL RECUPERADO**
MEDLINE/ Pubmed	P	Profissionais da saúde	#1	"Personnel, Health" OR "Health Care Providers" OR "Health Care Provider" OR "Provider, Health Care" OR "Healthcare Providers" OR "Healthcare Provider" OR "Provider, Healthcare" OR "Healthcare Workers" OR "Healthcare Worker" OR "Health Care	332,736

				Professionals" OR "Health Care Professional" OR "Professional, Health Care" OR "Health personnel"	
	C	Auriculoterapia	#2	auriculot* OR acupuntura auricular OR "ear acupuncture" OR "auricular acupuncture" OR "acupuncture earlobe" OR "auriculo-acupuncture" OR auriculoacupuncture	1,698
	C	Formação	#3	Credentialing OR "Training Program" OR Teaching OR "Inservice Training" OR “Training, Inservice” OR "intellectual training" OR "training support" OR workshop OR education OR “On-the-Job Training” OR “On the Job Training” OR “Training, On-the-Job” OR “Orientation Programs, Employee” OR “Employee Orientation Program” OR “Orientation Program, Employee” OR “Program, Employee Orientation” OR “Programs, Employee Orientation” OR “Employee Orientation Programs” OR “Training Programs” OR “Program, Training” OR “Programs, Training” OR “Training Program” OR “Educational Activities” OR “Activities, Educational” OR “Activity, Educational” OR “Educational Activity” OR “Literacy Programs” OR “Literacy Program” OR “Program, Literacy” OR “Programs, Literacy” OR “Training Techniques” OR “Training Technique” OR “Technique, Training” OR “Techniques, Training” OR Pedagogy OR Pedagogies OR “Teaching Method” OR “Academic Training” OR “Training, Academic” OR “Training Activities” OR “Training Activity” OR “Techniques, Educational” OR “education servisse” OR “educational measurement”	2,640,998
	C	Em qualquer contexto		-	-
	**Combinando estratégias**				
	#1#2#3	(("Personnel, Health" OR "Health Care Providers" OR "Health Care Provider" OR "Provider, Health Care" OR "Healthcare Providers" OR "Healthcare Provider" OR "Provider, Healthcare" OR "Healthcare Workers" OR "Healthcare Worker" OR "Health Care Professionals" OR "Health Care Professional" OR "Professional, Health Care" OR "Health personnel") AND (auriculot* OR acupuntura auricular OR "ear acupuncture" OR "auricular acupuncture" OR "acupuncture earlobe" OR "auriculo-acupuncture" OR auriculoacupuncture)) AND (Credentialing OR "Training Program" OR Teaching OR "Inservice Training" OR "Training, Inservice" OR "intellectual training" OR "training support" OR workshop OR education OR "On-the-Job Training" OR "On the Job Training" OR "Training, On-the-Job" OR "Orientation Programs, Employee" OR "Employee Orientation Program" OR "Orientation Program, Employee" OR "Program, Employee Orientation" OR "Programs, Employee Orientation" OR "Employee Orientation Programs" OR "Training Programs" OR "Program, Training" OR "Programs, Training" OR "Training Program" OR "Educational Activities" OR "Activities, Educational" OR "Activity, Educational" OR "Educational Activity" OR "Literacy Programs" OR "Literacy Program" OR "Program, Literacy" OR "Programs, Literacy" OR "Training Techniques" OR			12

		"Training Technique" OR "Technique, Training" OR "Techniques, Training" OR Pedagogy OR Pedagogies OR "Teaching Method" OR "Academic Training" OR "Training, Academic" OR "Training Activities" OR "Training Activity" OR "Techniques, Educational" OR "education servisse" OR "educational measurement")	
	#2#3	(auriculot* OR "ear acupuncture" OR "auricular acupuncture" OR "acupuncture earlobe" OR auriculoacupuncture) AND (Teaching OR education)	111
	**Estratégia escolhida**		
	#2#3	(auriculot* OR "ear acupuncture" OR "auricular acupuncture" OR "acupuncture earlobe" OR auriculoacupuncture) AND (Teaching OR education)	111

Teste de combinação dos descritores com os operadores adequados (OR, AND, AND NOT) para EMBASE:

**CONTROLE DE ESTRATÉGIAS REALIZADAS 18/08/2024**					
**FONTES**	**ACRÔNIMO**	**ELEMENTO DA PERGUNTA**	**#**	**ESTRATÉGIA**	**TOTAL RECUPERADO**
EMBASE (elsevier)	P	Profissionais da saúde	#1	"Personnel, Health" OR "Health Care Providers" OR "Health Care Provider" OR "Provider, Health Care" OR "Healthcare Providers" OR "Healthcare Provider" OR "Provider, Healthcare" OR "Healthcare Workers" OR "Healthcare Worker" OR "Health Care Professionals" OR "Health Care Professional" OR "Professional, Health Care" OR "Health personnel"	2,246,185
	C	Auriculoterapia	#2	auriculot* OR “acupuntura auricular” OR auriculotherap* OR "ear acupuncture" OR "auricular acupuncture" OR "auriculo-acupuncture" OR auriculoacupuncture	2,599
	C	Formação	#3	Credentialing OR "Training Program" OR Teaching OR "Inservice Training" OR “Training, Inservice” OR "intellectual training" OR "training support" OR workshop OR education OR “On-the-Job Training” OR “On the Job Training” OR “Training, On-the-Job” OR “Orientation Programs, Employee” OR “Employee Orientation Program” OR “Orientation Program, Employee” OR “Program, Employee Orientation” OR “Programs, Employee Orientation” OR “Employee Orientation Programs” OR “Training Programs” OR “Program, Training” OR “Programs, Training” OR “Training Program” OR “Educational Activities” OR “Activities, Educational” OR “Activity, Educational” OR “Educational Activity” OR “Literacy Programs” OR “Literacy Program” OR “Program, Literacy” OR “Programs, Literacy” OR “Training Techniques” OR “Training Technique” OR “Technique, Training”	3,371,591

				OR “Techniques, Training” OR Pedagogy OR Pedagogies OR “Teaching Method” OR “Academic Training” OR “Training, Academic” OR “Training Activities” OR “Training Activity” OR “Techniques, Educational” OR “education servisse” OR “educational measurement”	
	C	Em qualquer contexto		-	-
	**Combinando estratégias**				
	#1#2#3			('personnel, health' OR 'health care providers' OR 'health care provider' OR 'provider, health care' OR 'healthcare providers' OR 'healthcare provider' OR 'provider, healthcare' OR 'healthcare workers' OR 'healthcare worker' OR 'health care professionals' OR 'health care professional' OR 'professional, health care' OR 'health personnel') AND (auriculot* OR 'acupuntura auricular' OR auriculotherap* OR 'ear acupuncture' OR 'auricular acupuncture' OR 'auriculo-acupuncture' OR auriculoacupuncture) AND (credentialing OR teaching OR 'inservice training' OR 'training, inservice' OR 'intellectual training' OR 'training support' OR workshop OR education OR 'on-the-job training' OR 'on the job training' OR 'training, on-the-job' OR 'orientation programs, employee' OR 'employee orientation program' OR 'orientation program, employee' OR 'program, employee orientation' OR 'programs, employee orientation' OR 'employee orientation programs' OR 'training programs' OR 'program, training' OR 'programs, training' OR 'training program' OR 'educational activities' OR 'activities, educational' OR 'activity, educational' OR 'educational activity' OR 'literacy programs' OR 'literacy program' OR 'program, literacy' OR 'programs, literacy' OR 'training techniques' OR 'training technique' OR 'technique, training' OR 'techniques, training' OR pedagogy OR pedagogies OR 'teaching method' OR 'academic training' OR 'training, academic' OR 'training activities' OR 'training activity' OR 'techniques, educational' OR 'education servisse' OR 'educational measurement')	8
	#2#3			(auriculot* OR auriculotherap* OR 'ear acupuncture' OR 'auricular acupuncture' OR 'auriculo-acupuncture' OR auriculoacupuncture) AND (credentialing OR teaching OR workshop OR education OR 'training program')	157
	**Estratégia escolhida**				
	#2#3			(auriculot* OR auriculotherap* OR 'ear acupuncture' OR 'auricular acupuncture' OR 'auriculo-acupuncture' OR auriculoacupuncture) AND (credentialing OR teaching OR workshop OR education OR 'training program')	157

Teste de combinação dos descritores com os operadores adequados (OR, AND, AND NOT) para SCOPUS:

**CONTROLE DE ESTRATÉGIAS REALIZADAS 18/08/2024**					
**FONTES**	**ACRÔNIMO**	**ELEMENTO DA PERGUNTA**	**#**	**ESTRATÉGIA**	**TOTAL RECUPERADO**
SCOPUS	P	Profissionais da saúde	#1	"Personnel, Health" OR "Health Care Providers" OR "Health Care Provider" OR "Provider, Health Care" OR "Healthcare Providers" OR "Healthcare Provider" OR "Provider, Healthcare" OR "Healthcare Workers" OR "Healthcare Worker" OR "Health Care	359,591

				Professionals" OR "Health Care Professional" OR "Professional, Health Care" OR "Health personnel"	
	C	Auriculoterapia	#2	(auriculot* OR "ear acupuncture" OR "auricular acupuncture" OR "auriculo-acupuncture" OR auriculoacupuncture)	2,492
	C	Formação	#3	Credentialing OR "Training Program" OR Teaching OR "Inservice Training" OR “Training, Inservice” OR "intellectual training" OR "training support" OR workshop OR education OR “On-the-Job Training” OR “On the Job Training” OR “Training, On-the-Job” OR “Orientation Programs, Employee” OR “Employee Orientation Program” OR “Orientation Program, Employee” OR “Program, Employee Orientation” OR “Programs, Employee Orientation” OR “Employee Orientation Programs” OR “Training Programs” OR “Program, Training” OR “Programs, Training” OR “Training Program” OR “Educational Activities” OR “Activities, Educational” OR “Activity, Educational” OR “Educational Activity” OR “Literacy Programs” OR “Literacy Program” OR “Program, Literacy” OR “Programs, Literacy” OR “Training Techniques” OR “Training Technique” OR “Technique, Training” OR “Techniques, Training” OR Pedagogy OR Pedagogies OR “Teaching Method” OR “Academic Training” OR “Training, Academic” OR “Training Activities” OR “Training Activity” OR “Techniques, Educational” OR “education servisse” OR “educational measurement”	3,430,771
	C	Em qualquer contexto		-	-
	**Combinando estratégias**				
	#1#2#3	(TITLE-ABS-KEY ("personenel, health" OR "Health Care Providers" OR "Health Care Provider" OR "Provider, Health Care" OR "Healthcare Providers" OR "Healthcare Provider" OR "Provider, Healthcare" OR "Healthcare Workers" OR "Healthcare Worker" OR "health care professioneals" OR "health care professioneal" OR "professioneal, health care" OR "health personenel") AND TITLE-ABS-KEY ((auriculot* OR "ear acupuncture" OR "auricular acupuncture" OR "auriculo-acupuncture" OR auriculoacupuncture)) AND TITLE-ABS-KEY (Credentialing OR "Training Program" OR Teaching OR "Inservice Training" OR "Training, Inservice" OR "intellectual training" OR "training support" OR workshop OR education OR "one-the-job training" OR "one the job training" OR "training, one-the-job" OR "orientatione programs, employee" OR "employee orientatione program" OR "orientatione program, employee" OR "program, employee orientatione" OR "programs, employee orientatione" OR "employee orientatione programs" OR "Training Programs" OR "Program, Training" OR "Programs, Training" OR "Training Program" OR "educationeal activities" OR "activities, educationeal" OR "activity, educationeal" OR "educationeal activity" OR "Literacy Programs" OR "Literacy Program" OR "Program, Literacy" OR "Programs, Literacy" OR "Training Techniques" OR "Training Technique" OR "Technique, Training" OR "Techniques, Training" OR Pedagogy OR pedagogics OR "Teaching Method" OR "Academic Training" OR "Training, Academic" OR "Training Activities" OR "Training Activity" OR "techniques, educationeal" OR "educatione servisse" OR "educationeal measurement"))			10

	#2#3	(TITLE-ABS-KEY (teaching OR workshop OR education OR "Training Program") AND TITLE-ABS-KEY ((auriculot* OR "ear acupuncture" OR "auricular acupuncture" OR "auriculo-acupuncture" OR auriculoacupuncture)))	90
	**Estratégia escolhida**		
	#2#3	(TITLE-ABS-KEY (teaching OR workshop OR education OR "Training Program") AND TITLE-ABS-KEY ((auriculot* OR "ear acupuncture" OR "auricular acupuncture" OR "auriculo-acupuncture" OR auriculoacupuncture)))	90

Teste de combinação dos descritores com os operadores adequados (OR, AND, AND NOT) para Web Of Science:

**CONTROLE DE ESTRATÉGIAS REALIZADAS 18/08/2024**					
**FONTES**	**ACRÔNIMO**	**ELEMENTO DA PERGUNTA**	**#**	**ESTRATÉGIA**	**TOTAL RECUPERADO**
Web of Science	P	Profissionais da saúde	#1	"Personnel, Health" OR "Health Care Providers" OR "Health Care Provider" OR "Provider, Health Care" OR "Healthcare Providers" OR "Healthcare Provider" OR "Provider, Healthcare" OR "Healthcare Workers" OR "Healthcare Worker" OR "Health Care Professionals" OR "Health Care Professional" OR "Professional, Health Care" OR "Health personnel"	137,307
	C	Auriculoterapia	#2	(auriculot* OR "ear acupuncture" OR "auricular acupuncture" OR "auriculo-acupuncture" OR auriculoacupuncture OR auriculopuncture)	1,428
	C	Formação	#3	Credentialing OR "Training Program" OR Teaching OR "Inservice Training" OR “Training, Inservice” OR "intellectual training" OR "training support" OR workshop OR education OR “On-the-Job Training” OR “On the Job Training” OR “Training, On-the-Job” OR “Orientation Programs, Employee” OR “Employee Orientation Program” OR “Orientation Program, Employee” OR “Program, Employee Orientation” OR “Programs, Employee Orientation” OR “Employee Orientation Programs” OR “Training Programs” OR “Program, Training” OR “Programs, Training” OR “Training Program” OR “Educational Activities” OR “Activities, Educational” OR “Activity, Educational” OR “Educational Activity” OR “Literacy Programs” OR “Literacy Program” OR “Program, Literacy” OR “Programs, Literacy” OR “Training Techniques” OR “Training Technique” OR “Technique, Training” OR “Techniques, Training” OR Pedagogy OR Pedagogies OR “Teaching Method” OR “Academic Training” OR “Training, Academic” OR “Training Activities” OR “Training	7,576,582

				Activity” OR “Techniques, Educational” OR “education servisse” OR “educational measurement”	
	C	Em qualquer contexto		-	-
	**Combinando estratégias**				
	#1#2#3	"Personnel, Health" OR "Health Care Providers" OR "Health Care Provider" OR "Provider, Health Care" OR "Healthcare Providers" OR "Healthcare Provider" OR "Provider, Healthcare" OR "Healthcare Workers" OR "Healthcare Worker" OR "Health Care Professionals" OR "Health Care Professional" OR "Professional, Health Care" OR "Health personnel" (All Fields) AND (auriculot* OR "ear acupuncture" OR "auricular acupuncture" OR "auriculo-acupuncture" OR auriculoacupuncture OR auriculopuncture) (All Fields) AND Credentialing OR "Training Program" OR Teaching OR "Inservice Training" OR “Training, Inservice” OR "intellectual training" OR "training support" OR workshop OR education OR “On-the-Job Training” OR “On the Job Training” OR “Training, On-the-Job” OR “Orientation Programs, Employee” OR “Employee Orientation Program” OR “Orientation Program, Employee” OR “Program, Employee Orientation” OR “Programs, Employee Orientation” OR “Employee Orientation Programs” OR “Training Programs” OR “Program, Training” OR “Programs, Training” OR “Training Program” OR “Educational Activities” OR “Activities, Educational” OR “Activity, Educational” OR “Educational Activity” OR “Literacy Programs” OR “Literacy Program” OR “Program, Literacy” OR “Programs, Literacy” OR “Training Techniques” OR “Training Technique” OR “Technique, Training” OR “Techniques, Training” OR Pedagogy OR Pedagogies OR “Teaching Method” OR “Academic Training” OR “Training, Academic” OR “Training Activities” OR “Training Activity” OR “Techniques, Educational” OR “education servisse” OR “educational measurement” (All Fields)			6
	#2#3	(auriculot* OR "ear acupuncture" OR "auricular acupuncture" OR "auriculo-acupuncture" OR auriculoacupuncture OR auriculopuncture) (All Fields) AND "Training Program" OR Teaching OR workshop OR education (All Fields)			122
	**Estratégia escolhida**				
	#2#3	(auriculot* OR "ear acupuncture" OR "auricular acupuncture" OR "auriculo-acupuncture" OR auriculoacupuncture OR auriculopuncture) (All Fields) AND "Training Program" OR Teaching OR workshop OR education (All Fields)			122

SELEÇÃO DOS ESTUDOS

A equipe de revisão será composta por: eu (1ª revisora) e outra doutoranda do grupo de pesquisa (2ª revisora), ainda, a terceira revisora será a orientadora do estudo, professora Maria Denise Schimith.

Como ferramenta para seleção (teste piloto e treinamento), será utilizado o quadro abaixo:

<table>
<tr><td colspan="7">Título da revisão: FORMAÇÃO EM AURICULOTERAPIA: protocolo de revisão de escopo.

Pergunta: COMO ESTÁ OCORRENDO A FORMAÇÃO/QUALIFICAÇÃO DE PROFISSIONAIS DA SAÚDE EM AURICULOTERAPIA?</td></tr>
<tr><td colspan="7">CHECK LIST DE INCLUSÃO</td></tr>
<tr><td>Tipos de estudos</td><td colspan="6">estudos primários, relatos de experiência, editoriais, artigos de opinião, artigos de reflexão, manuais, diretrizes ou guidelines.</td></tr>
<tr><td>Idiomas</td><td colspan="6">sem restrição de idioma</td></tr>
<tr><td>Recorte temporal</td><td colspan="6">sem recorte temporal</td></tr>
<tr><td>População</td><td colspan="6">estudos com qualquer profissional da saúde que possua alguma formação em auriculoterapia ou que relate o processo de formação dos mesmos</td></tr>
<tr><td>Conceito</td><td colspan="6">estudos que abordem qualquer tipo de formação em auriculoterapia ou acupuntura auricular, seja aperfeiçoamento, qualificação profissional, minicurso, curso de extensão, treinamento, pós-graduação, formação informal ou autodeclarada.</td></tr>
<tr><td>Contexto</td><td colspan="6">Realizados em qualquer contexto</td></tr>
<tr><td colspan="7">FERRAMENTA DE SELEÇÃO</td></tr>
<tr><td>IDENTIFICADOR</td><td>REFERÊNCIA</td><td colspan="3">SELECIONADO</td><td colspan="2">NÃO SELECIONADO</td></tr>
<tr><td>E1, E2, E3...</td><td>-</td><td>TIPO DE FORMAÇÃO</td><td>PAÍS</td><td>PROFISSIONAIS DA SAÚDE (QUAIS?)</td><td>NÃO RESPONDE À PERGUNTA</td><td>DUPLICADO</td></tr>
<tr><td></td><td></td><td></td><td></td><td></td><td></td><td></td></tr>
</table>

Para o gerenciamento das referências será utilizado o EndNote e Rayyan. O fluxograma para apresentar o processo de revisão será o PRISMA-ScR.

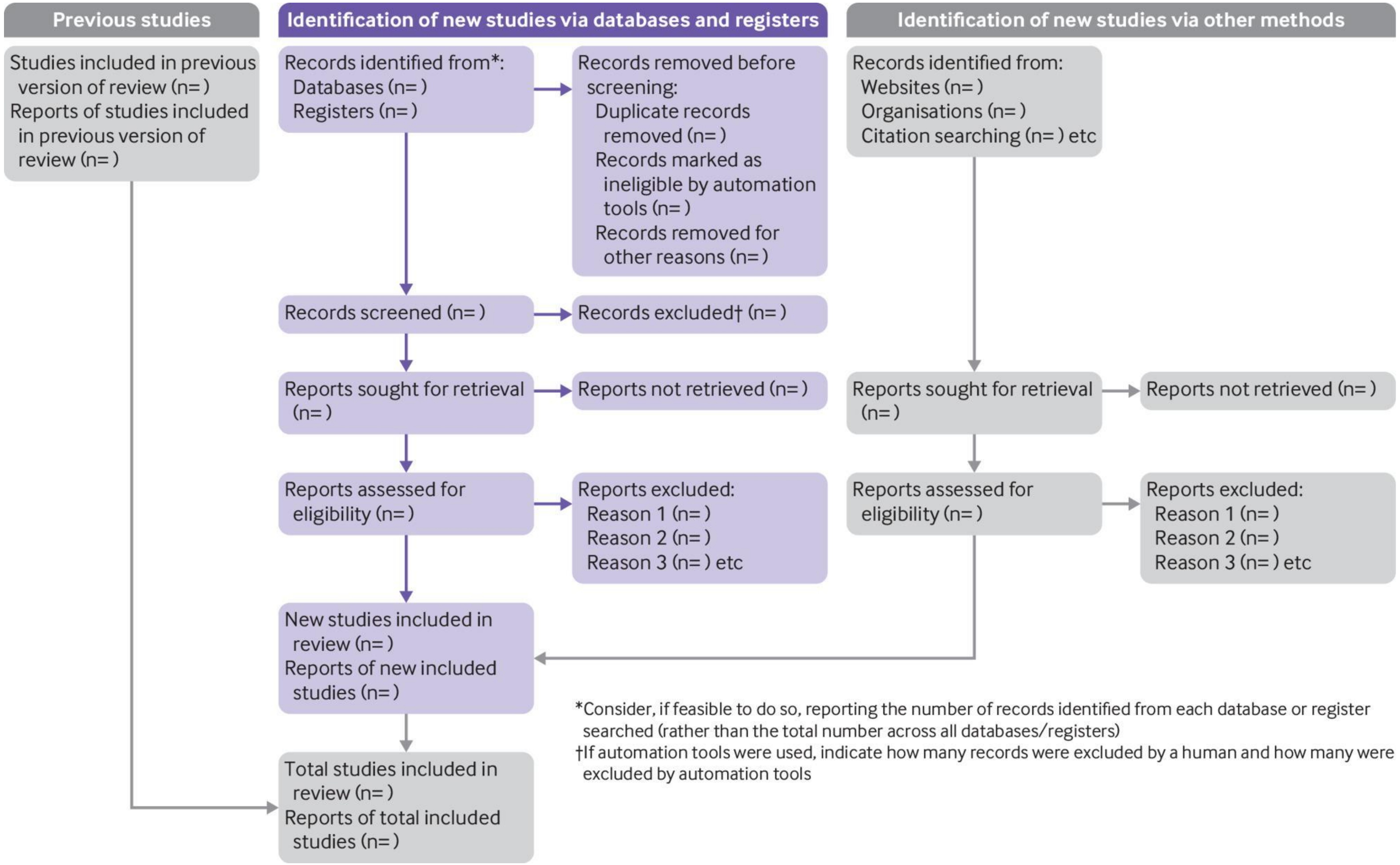

Fonte: Page M J, McKenzie J E, Bossuyt P M, Boutron I, Hoffmann T C, Mulrow C D et al. The PRISMA 2020 statement: an updated guideline for reporting systematic reviews BMJ 2021; 372 :n71 doi:10.1136/bmj.n71

EXTRAÇÃO DE DADOS

Irá ocorrer por meio do instrumento de extração de dados abaixo:

**Código**	E1	E2	E3
**Referência**			
**País**			
**Área do conhecimento**			
**Instituição de ensino**			
**Idioma**			
**População**			
**Objetivo**			
**Tipo de formação**			
**Duração/carga horária teórica**			
**Duração/carga horária prática**			
**Conteúdo programático**			
**Modalidade (online/presencial/híbrido)**			

REFERÊNCIAS

Page M J, McKenzie J E, Bossuyt P M, Boutron I, Hoffmann T C, Mulrow C D et al. The PRISMA 2020 statement: an updated guideline for reporting systematic reviews BMJ 2021; 372 :n71 doi:10.1136/bmj.n71

Bleck RR, Gold MA, Westhoff CL. Training hour requirements to provide acupuncture in the United States. Acupunct Med. 2021 Aug;39(4):327-333. doi: 10.1177/0964528420939576.

Tesser CD, Moré AOO, Santos MC, da Silva EDC, Farias FTP, Botelho LJ. Auriculotherapy in primary health care: A large-scale educational experience in Brazil. J Integr Med. 2019 Jul;17(4):302-309. doi: 10.1016/j.joim.2019.03.007.

WICKERT, D. C. .; DALLEGRAVE, D. .; PIEXAK, D. R. .; MELLO, M. C. V. A. de .; CORCINI, L. M. C. da S. .; SCHIMITH, M. D. . Práticas integrativas e complementares, perfil e cuidados de enfermeiras(os) às pessoas com hipertensão: estudo misto. Revista Latino-Americana de Enfermagem, [S. l.], v. 31, p. e3916, 2023. DOI: 10.1590/1518-8345.6287.3916.

Silva, P. H. B. da ., Barros, L. C. N. de ., Barros, N. F. de ., Teixeira, R. A. G., & Oliveira, E. S. F. de .. (2021). Formação profissional em Práticas Integrativas e Complementares: o sentido atribuído por trabalhadores da Atenção Primária à Saúde. Ciência & Saúde Coletiva, 26(2), 399–408. https://doi.org/10.1590/1413-81232021262.40732020.

EUROPEAN CENTRE OF DEVELOPMENTE OF VOCATIONAL TRAINEING. Terminology of European Education and Training Policy. 2° editions. Luxembourg: Publications Office of the European Union. 2014. Disponível em: https://www.cedefop.europa.eu/files/4117_en.pdf. Acesso em 17 jul. 2023.
