# Supplementary material for: Characteristics of teaching auriculotherapy/auricular acupuncture to healthcare professionals worldwide: a scoping review
Source: Rev Bras Enferm. 2025 Nov 3;78(4):e20240512. doi: 10.1590/0034-7167-2024-0512 (PMC12584949; doi:10.1590/0034-7167-2024-0512)
Supplement: Translation

# Translation: Características do ensino da auriculoterapia/acupuntura auricular para profissionais da saúde em nível mundial: *scoping review*

**DOI:** 10.1590/0034-7167-2024-0512pt

**Authors:** Daiana Cristina Wickert, Daniela Dallegrave, Oclaris Lopes Munhoz, Fernanda dos Santos Trombini, Maria Denise Schimith

**Affiliations:** I Universidade Federal de Santa Maria. Santa Maria, Rio Grande do Sul, Brasil; II Universidade Federal do Rio Grande do Sul. Porto Alegre, Rio Grande do Sul, Brasil; III Universidade Federal de Santa Maria. Palmeira das Missões, Rio Grande do Sul, Brasil

**Keywords:** Auriculoterapia, Acupuntura Auricular, Pessoal de Saúde, Ensino, Educação

## Abstract

**Objetivos:**

mapear as características da formação de profissionais da saúde em auriculoterapia/acupuntura auricular em nível mundial.

**Métodos:**

revisão de escopo, de acordo com o JBI, desenvolvida em oito fontes de informação. Foram incluídos estudos que abordaram a formação em auriculoterapia, independentemente do delineamento. Procedeu-se à análise e síntese descritiva e narrativa.

**Resultados:**

incluíram-se 14 produções, sendo a maioria artigos (n=9; 64,3%), disponíveis em inglês (n=10; 71,4%). Nenhum curso afirmou ser totalmente *online*, com carga horária entre 3 e 228 horas. Brasil e França formam auriculoterapeutas. Estados Unidos da América, Austrália e Ucrânia direcionam a formação com ênfase em protocolos específicos. Quanto aos formadores, Brasil e Austrália possuem equipes multiprofissionais elaborando e ministrando os cursos/aulas.

**Considerações Finais:**

identificou-se que a formação ocorre de forma distinta no mundo, carecendo de requisitos mínimos definidos. Não foram recuperadas investigações que avaliaram diretrizes de formação, pesquisas necessárias para o avanço na oferta de cuidados.

## INTRODUÇÃO

As medicinas alternativas e complementares (MAC), assim reconhecidas na literatura científica internacional (em inglês, *complementary and alternative medicine*), ou, ainda, designadas pela Organização Mundial da Saúde (OMS) como medicinas tradicionais, complementares e integrativas (MTCI), são práticas, produtos e saberes utilizados por diferentes povos e culturas do mundo. O modelo de cuidado das MTCI considera o indivíduo em sua singularidade e integralidade, bem como o contexto sociocultural em que vive, sendo, assim, um modelo de humanização do cuidado^(1)^. As MTCI podem ser aplicadas por terapeutas com formação e por profissionais de saúde capacitados^(2)^.

Entre o escopo das MTCI, tem-se a auriculoterapia, que em 1990 foi reconhecida pela OMS como terapia de microssistema para promoção e manutenção da saúde no tratamento de diversas enfermidades, realizada por estímulo de pontos da orelha externa^(3)^. As evidências não possuem um consenso acerca da origem da terapia auricular, que é utilizada há milhares de anos em diversos países; no entanto, pode-se afirmar que o maior desenvolvimento da técnica e os primeiros documentos registrados são da China^(4)^.

A primeira documentação não chinesa sobre acupuntura auricular remonta ao antigo Egito, onde o tratamento de problemas ginecológicos foi mencionado no Papiro *Ebers* em 1500 a.C^(5)^. Assim, considerando a história da auriculoterapia, quando se pensa em prática clínica e formação de profissionais, não se podem ignorar as três vertentes mundiais, como a reflexologia da orelha, a auriculoterapia segundo a medicina tradicional chinesa (MTC) e a neurofisiologia, que coexistem entre si com suas diferenças e semelhanças.

Ao considerar a inexistência de um consenso entre os mapas auriculares^(3)^, pode-se deduzir que a formação dos profissionais que atuam com auriculoterapia e/ou acupuntura auricular tampouco possui critérios bem definidos nos diferentes países do mundo. A Medicina Tradicional e Complementar é amplamente utilizada em todo o mundo, com 170 dos 194 estados-membros da OMS reconhecendo seu uso. No entanto, em 2018 [informação mais recente disponível], apenas 78 países indicaram que regulamentam os praticantes. O apoio da OMS à regulamentação inclui a publicação de dez referências (*benchmarks*) para treinamento e sete referências para prática^(6)^.

O *benchmark* da OMS sobre acupuntura, publicado em 1999 e atualizado em 2020, é resultado de um rigoroso processo de mapeamento e revisão dos padrões de formação de especialistas dessa abordagem em saúde. O documento é dividido em: (1) introdução; (2) categorias de treinamento; (3) níveis de treinamento; (4) componentes e requisitos para treinamento completo; (5) componentes e requisitos para formação adaptada. Essas cinco partes constituem um conjunto completo de referências para o treinamento em acupuntura; assim, o documento pode servir de base para estabelecer sistemas de treinamento, exame e licenciamento que apoiam a prática qualificada da acupuntura^(7)^.

No entanto, o documento em questão não aborda de forma específica a formação em auriculoterapia e/ou acupuntura auricular, e inexiste um *benchmark* direcionado para tal. Dessa forma, considerando que a formação em auriculoterapia pode se dar dentro da formação de especialização em acupuntura, mas também de forma separada, apresenta-se a necessidade de conhecer os parâmetros de referência existentes.

Nesse sentido, a presente revisão de escopo demonstra como a formação em auriculoterapia e/ou acupuntura auricular vem ocorrendo no mundo, o que pode fornecer subsídios para a elaboração de parâmetros mínimos em pesquisas futuras, assim como para cursos relacionados à prática.

## OBJETIVOS

Mapear as características da formação de profissionais da saúde em auriculoterapia/acupuntura auricular em nível mundial.

## MÉTODOS

### Aspectos éticos

Todos os princípios éticos foram respeitados. A veracidade dos resultados foi mantida e os estudos foram devidamente citados. No entanto, por se tratar de estudo de revisão, a apreciação do Comitê de Ética em Pesquisa foi dispensada.

### Planejamento e desenho do estudo

Foi realizada uma busca preliminar nas fontes PROSPERO, COCHRANE, JBI *Evidence Synthesis*, PubMed e *Open Science Framework* (OSF) em 10 de março de 2024, não sendo identificada nenhuma revisão que responda ao problema em questão. Assim, o protocolo de revisão foi registrado na plataforma OSF, sob número de registro DOI 10.17605/OSF.IO/5J86R, o qual pode ser acessado na íntegra no *link* https://osf.io/5j86r/.

Trata-se de revisão de escopo (*scoping review*) elaborada de acordo com as etapas metodológicas do JBI^(8)^ e relatada em consonância com as recomendações do *Preferred Reporting Items for Systematic Reviews and Meta-Analyses Extension for Scoping Reviews* (PRISMA-ScR)^(9)^. Nove etapas foram seguidas^(8)^: (1) definir e alinhar o objetivo e pergunta; (2) desenvolver e alinhar os critérios de inclusão com o objetivo e pergunta; (3) descrever a abordagem planejada para busca, seleção, extração e mapeamento de evidências; (4) procurar pelas evidências; (5) selecionar a evidência; (6) extrair as evidências; (7) analisar os resultados; (8) apresentar os resultados; e (9) resumir as evidências em relação ao propósito da revisão, tirar conclusões e observar quaisquer implicações das conclusões.

### Identificação da questão de revisão

A questão de revisão foi estruturada com uso do mnemônico PCC (População, Conceito e Contexto), sendo População os profissionais da saúde, Conceito, formação/qualificação em auriculoterapia ou acupuntura auricular, e Contexto, todos os contextos em que há a formação. Elaborou-se, assim, a seguinte questão de revisão: como ocorre a formação/qualificação de profissionais da saúde em auriculoterapia ou acupuntura auricular, independentemente do contexto?

### Critérios de seleção e identificação dos estudos relevantes

Foram incluídos estudos primários, relatos de experiência, editoriais, artigos de opinião, artigos de reflexão, teses, dissertações, manuais, diretrizes ou *guidelines*, sem restrição de idioma, recorte temporal ou delimitação geográfica. Foram excluídos estudos realizados simultaneamente com estudantes de graduação e profissionais da saúde em que os dados não puderam ser separados para análise. Não se definiu recorte de idioma e temporal, assim como produções duplicadas foram consideradas uma vez.

As estratégias de busca foram inseridas nos sítios específicos de cada fonte no dia 18 de agosto de 2024 via Portal da Coordenação de Aperfeiçoamento de Pessoal de Nível Superior (CAPES), por meio de acesso remoto da Comunidade Acadêmica Federada (CAFe) da Universidade Federal de Santa Maria. Foram utilizadas cinco bases de dados: EMBASE (Elsevier), *Web of Science* (WoS), *Medical Literature Analysis and Retrieval System Online* (MEDLINE), via PubMed, Scopus (Elsevier), e Modelos de Saúde e Medicamentos Tradicionais, Complementares e Integrativos nas Américas (MOSAICO - VHL TCIM), via Biblioteca Virtual em Saúde (BVS). Ademais, foram realizadas buscas na literatura cinzenta: Biblioteca Digital Brasileira de Teses e Dissertações (BDTD), Portal de Teses e Dissertações da CAPES e *OpenGrey*. A lista de referências dos estudos incluídos também foi analisada.

### Estratégias de busca, triagem e seleção dos estudos

Foram definidas estratégias de busca específicas para cada fonte de dados, após teste piloto na MEDLINE e amplo mapeamento de termos controlados e sinônimos no *Medical Subject Headings Section* (MeSH), *Emtree terms* e nos Descritores em Ciências da Saúde (DeCS), em espanhol, inglês e português. As estratégias foram elaboradas e discutidas na elaboração do protocolo de revisão durante a disciplina de um programa de doutorado. Foi realizada exaustiva testagem das estratégias nas bases, visando uma busca de alta sensibilidade exequível que respeitasse as especificidades de cada fonte e que recuperasse o número máximo de artigos que respondessem à pergunta de revisão. O mapeamento de termos e o teste de combinação dos descritores com os operadores booleanos adequados podem ser visualizados no Quadro 1.

**Quadro 1:** Estratégias de busca, Santa Maria, Rio Grande do Sul, Brasil, 2024

Base	Estratégias de busca (18/08/2024)
MOSAICO	auriculoterapia *OR* “acupuntura auricular” *OR* “Acupuntura na Orelha” *OR auriculoacupuntura OR auriculopuntura OR* “*acupuntura en la oreja*” *OR* “*Acupuncture*, *Ear*” *OR* “*auricular acupuncture*” OR Auriculot*
MEDLINE	(auriculot* *OR* “*ear acupuncture*” *OR* “*auricular acupuncture*” *OR* “*acupuncture earlobe*” *OR auriculoacupuncture*) *AND* (*Teaching OR education*)
EMBASE	(auriculot* OR auriculotherap* *OR ‘ear acupuncture’ OR ‘auricular acupuncture’ OR ‘auriculo*-*acupuncture’ OR auriculoacupuncture*) *AND* (*credentialing OR teaching OR workshop OR education OR ‘training program’*)
Scopus	( *TITLE*-*ABS*-*KEY* ( ( auriculot* *OR* “*ear acupuncture*” *OR* “*auricular acupuncture*” *OR* “*auriculo*-*acupuncture*” *OR auriculoacupuncture* ) ) *AND TITLE*-*ABS*-*KEY* ( *teaching OR workshop OR education OR* “*Training Program*” ) )
*Web of Science*	(auriculot* *OR* “*ear acupuncture*” *OR* “*auricular acupuncture*” *OR* “*auriculo*-*acupuncture*” *OR auriculoacupuncture OR auriculopuncture*) (*All Fields*) *AND* “*Training Program*” *OR Teaching OR workshop OR education* (*All Fields*)
Biblioteca Digital Brasileira de Teses e Dissertações	auriculoterapia *OR* “acupuntura auricular” *OR* “Acupuntura na Orelha” *OR* auriculoacupuntura
Portal de Teses e Dissertações da CAPES	auriculoterapia *OR* “acupuntura auricular” *OR* “Acupuntura na Orelha” *OR* auriculoacupuntura
*OpenGrey*	auriculot* *OR* “*ear acupuncture*” *OR* “*auricular acupuncture*” *OR* “*acupuncture earlobe*” *OR* “*auriculo*-*acupuncture*” *OR auriculoacupuncture*

Após as buscas, os resultados foram exportados para o gerenciador de referências *EndNote Web*, no qual foram excluídas as duplicatas. Em seguida, os resultados foram enviados ao aplicativo/*website Rayyan QCRI* para análise e seleção independente/cega, realizada por duas revisoras doutorandas e integrantes do grupo de pesquisa ao qual a produção está vinculada. Após as avaliações, em reunião de consenso, foram checadas as divergências, da qual a terceira revisora participou, porém não precisou intervir. Menciona-se que ocorreu primeiramente a leitura de títulos e resumos (etapa 1), sendo realizada uma leitura na íntegra em uma segunda seleção (etapa 2).

### Extração de dados

Esta etapa também foi conduzida por duas revisoras. Um instrumento específico foi elaborado no *Microsoft Excel*, sendo extraídas as informações sobre referência, tipo de documento, país, área do conhecimento, instituição de ensino, idioma, população, objetivo, tipo de formação, duração/carga horária, conteúdo programático e modalidade (*online*, presencial ou semipresencial).

### Análise dos dados

A análise dos dados foi realizada de forma descritiva e narrativa, de acordo com a similaridade dos resultados, que foram organizados em categorias. Ainda, procedeu-se ao uso de frequências absolutas e relativas, figuras e quadros.

## RESULTADOS

A partir das estratégias de busca, foi possível identificar 524 produções, sendo que, dessas, 211 estavam duplicadas. Assim, na fase de seleção por título e resumo, realizou-se a leitura de 313 produções. Dessas, 287 foram excluídas por não atenderem aos critérios de seleção. Na etapa seguinte, 26 registros foram elencados para a leitura na íntegra, dos quais cinco não estavam disponíveis (esgotadas as possibilidades de acesso), sendo excluídos da revisão. Logo, 21 produções foram lidas na íntegra, etapa em que nove foram excluídas. Nas buscas de literatura cinza e de listas de referências, 208 registros tiveram seus títulos e resumos lidos, sendo três acessados na íntegra, em que um foi excluído por não atender aos critérios de seleção. Assim, 14 registros constituíram a síntese das evidências (Figura 1).

**Figura 1:** Fluxograma PRISMA ScR de busca e seleção dos estudos, Santa Maria, Rio Grande do Sul, Brasil, 2024

A seguir, no Quadro 2, apresentam-se características referentes à população que realizou o curso, ao tipo de formação/titulação que receberam, à carga horária e modalidade de ensino dos cursos de formação em auriculoterapia, às instituições envolvidas na formação, e à sua localização geográfica (país).

**Quadro 2:** Características das formações descritas nos estudos que compõem a amostra, Santa Maria, Rio Grande do Sul, Brasil, 2024

Autores (ano)	População que realizou o curso	Tipo de formação/ titulação	Carga horária Modalidade de ensino	Instituições envolvidas na oferta das formações e país
Oyola-Santiago; Knopf; Robin; Harvey (2013)^(10)^	Sem informação	Especialistas em desintoxicação por acupuntura	- 70 horas - Presencial	*- The New School in New York* - EUA
King; Hickey; Connelly (2013)^(11)^	Prestadores de cuidado de saúde militar	Não especifica	- 4 a 8 horas - Presencial, com ensino didático e prática clínica, seguidos de um breve exame	- Centro de Acupuntura da Força Aérea localizado na Base Aérea de Andrews, em Maryland - EUA
Stanton; Rangon (2014)^(12)^	Médicos	*Diplôme Inter Universitaire* sob a orientação do Dr. David Alimi	- 228 horas aula (200 horas de teoria e 28 práticas) - Presencial, com duração de dois anos, distribuídos por sete fins de semana, de novembro a maio, anualmente, ministradas, geralmente, no *Institut Gustave Roussy*	*- Universities of Paris XIII and XI* - França
Ferreira (2016)^(13)^	Profissionais da saúde de nível superior	Educação permanente	- Curso 1: 8 horas; Curso 2: 80 horas - Curso 1: não especifica a modalidade; Curso 2: semipresencial	- UFSC - Brasil
Niemtzow; Baxter; Gallagher; Pock; Calabria; Drake et al. (2018)^(14)^	Prestadores de cuidados em saúde	*Acupuncture Training Across Clinical Settings* para aplicar BFA	- 4 horas aula - Presencial	- Força Aérea, Exército e Marinha - EUA
Haywood; Drake; Condie (2019)^(15)^	Médicos residentes	Treinamento para aplicar BFA	- 3 horas - Presencial, palestra didática ministrada por um instrutor certificado e depois uma demonstração prática	- Programa de Residência em Medicina Física e Reabilitação - EUA
Tesser; Moré; Santos; da Silva; Farias; Botelho (2019)^(16)^	Profissionais da saúde de nível superior e atuantes na APS	Curso semipresencial	- 75 horas de ensino a distância (cinco módulos sequenciais) e 5 horas presenciais - Semipresencial	- UFSC - Brasil
Jan (2020)^(17)^	Médicos	Curso semipresencial	- Curso 1: 4 horas (com mais 4 horas *online*), sobre corpo e BFA no 35º Encontro Científico de Medicina de Emergência Curso 2: 2 horas (com 2 horas extras, *online*), realizado no local do teste para funcionários do pronto-socorro que aplicaram apenas o BFA - Semipresencial	*- Australasian College for Emergency Medicine* - Austrália
Jan (2020)^(18)^	Médicos e enfermeiros de pronto-socorro	*Workshops*	- Não especifica a carga horária - *Online* e presenciais	*- Emergency Department, St. John of God Murdoch Hospital* - Austrália
Silva; Barros LCN de, Barros NF de; Teixeira; Oliveira (2021)^(19)^	- Curso 1: nutricionista e farmacêutica - Curso 2: farmacêutica - Curso 3: enfermeira	Capacitação	- Curso 1: 80 horas Curso 2: 40 horas Curso 3: 12 horas (8 horas teórica e 4 horas práticas) - Curso 1: parte teórica *online* e parte prática presencial; Curso 2: presencial; Curso 3: presencial	- UFSC, Secretaria Municipal de Saúde de Goiânia e Conselho Regional de Enfermagem de Goiás - Brasil
Hildebrand (2023)^(20)^	Médicos, psicólogos, fisioterapeutas, paramédicos militares e profissionais que se identificam com alguma ligação a aconselhamento e atividades terapêuticas	Educação continuada	- 14 horas - Presencial	*- Uzhhorod National University College of Medicine* - Ucrânia
Castle; Lukkahatai; Billing; Huang; Wu; Zhang et al (2023)^(21)^	Enfermeiros oncológicos	Educação continuada (*workshop*)	- 8 horas (5 horas didáticas e 3 práticas) - Presencial	*- Comprehensive Cancer Center in Maryland* - EUA
Hohenberger; Dallegrave (2016)^(22)^	Profissionais da saúde de nível superior e atuantes na APS	Capacitação	-75 horas de ensino a distância (cinco módulos sequenciais) e 5 horas de ensino presencial - Semipresencial	- UFSC - Brasil
Domingues (2019)^(23)^	Profissionais da saúde de nível superior e atuantes na APS	Capacitação	-75 horas de ensino a distância (cinco módulos sequenciais) e 5 horas de ensino presencial - Semipresencial	- UFSC - Brasil

*ID - identificação; APS - Atenção Primária à Saúde; BFA - Battlefield Acupuncture; EUA - Estados Unidos da América; UFSC - Universidade Federal de Santa Catarina.*

Entre as 14 (100%) produções incluídas, nove (64,3%) eram artigos^(11,12,14-16,18,19,21,22)^; duas (14,4%) eram dissertações^(13,23)^; uma era produção vinculada à apresentação oral^(17)^ (7,1%); e editorial^(20)^ (7,1%) e *brief report* ^(10)^ (7,1%) representaram um documento cada. Quanto ao idioma, dez (71,4%) estavam disponíveis em inglês^(10-12,14-18,20,21)^, e quatro (28,6%), em português^(13,19,22,23)^. No que tange ao ano de publicação, os documentos datam de 2013 a 2023, com predomínio em 2019 (n=3; 21,4%)^(15,16,23)^.

Destacaram-se, entre as populações das formações, os profissionais com nível superior da Atenção Primária à Saúde (APS) brasileira^(13,16,19,22,23)^, formados pelo curso ofertado pelo Ministério da Saúde em parceria com a Universidade Federal de Santa Catarina (UFSC). A formação tornou-os auriculoterapeutas, aptos a aplicar a técnica nas mais variadas situações, especialmente aquelas comuns em suas rotinas de trabalho no Sistema Único de Saúde (SUS). No entanto, outro público bem distinto do Brasil parece ser predominante nos EUA, que são profissionais que atuam em serviços de saúde nos cuidados de soldados e veteranos de guerra, principalmente voltados ao tratamento da dor^(11)^, utilizando um protocolo de cinco pontos auriculares denominado *Battlefield Acupuncture* (BFA)^(14,15)^, sendo que essa mesma formação aparece no continente europeu, na modalidade de educação continuada^(20)^, desenvolvida com médicos, psicólogos e fisioterapeutas na Ucrânia. Ainda, cabe destacar que, na Austrália, o curso em BFA foi direcionado a médicos e enfermeiros para que atuem no tratamento da dor e, consequentemente, redução do uso de opioides nas emergências ou prontos-socorros^(18,19)^.

Um único artigo dos EUA apresenta uma formação em educação continuada por meio de *workshop* realizada com enfermeiros para tratamento de dor oncológica^(21)^. Outro documento relata uma formação para o protocolo *National Acupuncture Detoxification Association* (NADA)^(10)^, certificando os concluintes como *acupuncture detoxification specialists* (ADSs), ou seja, aptos para aplicação de cinco pontos auriculares para tratamento voltados ao uso de drogas.

Quanto à carga horária, a formação de *Diplôme Interuniversitaire*, sob a orientação do Dr. David Alimi, na França, é a mais extensa, com 228 horas divididas em dois anos e oferecida de forma presencial. Cabe destacar que essa formação também é uma das que habilita o profissional médico a atuar de forma ampla, não direcionado a apenas um protocolo específico. Outro país com essa característica formativa é o Brasil, onde as formações em média possuem 80 horas, porém na modalidade semipresencial de ensino em curso do Ministério da Saúde em parceria com a UFSC. No entanto, os profissionais referem como ponto negativo a baixa carga horária, entendendo que 20 horas são pouco tempo para compreender a MTC^(19)^.

Cabe ressaltar que, apesar da formação voltada apenas ao protocolo NADA, os ADSs realizaram um curso com 70 horas presenciais. Já as formações em BFA tiveram uma média de 3 a 8 horas de formação, sendo na modalidade presencial nos EUA e Ucrânia, e semipresencial na Austrália. Os EUA divergem sobre quantas horas de treinamento em acupuntura são clinicamente necessárias para serem considerados praticantes dentro de seus respectivos escopos^(15)^.

No treinamento em acupressão do ponto auricular (APA)^(21)^, após conduzir o curso, os autores realizaram uma avaliação para examinar o conhecimento dos enfermeiros com base no conteúdo da oficina de APA. O teste de conhecimentos consistiu em 43 questões de múltipla escolha e sete questões abertas (a pontuação variou de 0 a 100). As questões abordavam a perspectiva teórica da auriculoterapia, terminologia anatômica da orelha externa relacionada ao corpo, procedimentos de diagnóstico auricular para localização dos pontos auriculares, pontos mestres dos efeitos do tratamento, pontos auriculares musculoesqueléticos e sensoriais, e como estimular os pontos auriculares. A pontuação média no teste de conhecimentos pós-curso foi de 93% (desvio padrão [DP] = 6,87), mostrando que 8 horas do treinamento podem ser consideradas adequadas para aplicação de APA para dor relacionada ao câncer.

Evidencia-se nos países cujos estudos foram recuperados para a presente revisão (EUA, Ucrânia e Austrália) um padrão de formações em protocolos específicos, como BFA e NADA, e não uma formação “geral” como auriculoterapeutas, para uso em diversas situações, como no Brasil^(13,16,19,22,23)^ e na França^(12)^, países em que a formação pareceu ser mais ampla.

A modalidade semipresencial foi observada apenas nas formações no Brasil e Austrália. Nenhum curso descrito afirmou ser totalmente *online*. Estudo^(23)^ que aborda a formação ofertada pela UFSC aponta que a capacitação é a iniciação na auriculoterapia, com indicação da necessidade de continuar a formação por meio de situações locais, como oficina ou grupos de estudos.

Na sequência, apresenta-se a caracterização geral das produções, categorizados em “Conteúdo programático dos cursos de auriculoterapia e/ou acupuntura auricular” e “Características dos formadores dos cursos de auriculoterapia e/ou acupuntura auricular”. O Quadro 3 sintetiza as informações que posteriormente serão aprofundadas para responder como as formações em auriculoterapia estão ocorrendo no mundo.

**Quadro 3:** Aspectos destacados nos conteúdos programáticos dos cursos de auriculoterapia, Santa Maria, Rio Grande do Sul, Brasil, 2024

Descrição do conteúdo programático identificado no estudo
Ensino do protocolo NADA, também conhecido como *ACU Detox*, que consiste na estimulação de cinco pontos auriculares (*Shen Men*, sistema nervoso simpático, fígado, rim e pulmão) para uso de drogas ou mesmo saúde mental^(10)^.
Enfatiza discussões gerais e específicas de casos clínicos de pacientes, envolvendo, principalmente, patologia e fisiopatologia, bem como diagnóstico e planejamento de tratamento. Primeiro ano: base biológica fundamental da auriculoterapia (incluindo elementos como embriologia, anatomia, genética e neurofisiologia). Segundo ano: aplicações clínicas da auriculoterapia pertencentes a todas as áreas da medicina (incluindo neurologia, cardiologia, endocrinologia, medicina pulmonar, pediatria, entre outros)^(12)^.
Revisar a história da acupuntura, o significado clínico e as evidências para seu uso; compreender os critérios de seleção de pacientes; fornecer informações educacionais aos pacientes sobre acupuntura; obter e documentar o consentimento; ganhar proficiência na técnica de agulhamento (por exemplo, localização e posicionamento da agulha); identificar, compreender e transmitir instruções e precauções pós-procedimento; obter o credenciamento apropriado. Foram utilizados palestras, vídeos, demonstrações e prática com orelhas de silicone e modelos vivos. A aprendizagem e a proficiência das competências dos formandos são avaliadas através da observação do formador e de exame escrito. Cada curso de formação termina com exame escrito. Uma avaliação do curso foi realizada na conclusão do treinamento^(14)^.
A sessão didática focou nas especificidades do protocolo BFA, incluindo segurança, técnica adequada de agulha, seleção de pacientes e cuidados com a agulha. Durante a demonstração prática, cada participante verificou seu conhecimento sobre o posicionamento correto de cinco agulhas BFA de acordo com o protocolo. Esta demonstração foi praticada primeiramente com orelhas de silicone seguida da colocação de agulhas nos demais residentes participantes^(15)^.
Os módulos incluíram os seguintes itens: (1) introdução às práticas integrativas; (2) reflexologia auricular; (3) introdução à MTC; (4) visão biomédica da auriculoterapia; e (5) auriculoterapia na APS. O material didático incluiu uma apostila para cada módulo, 14 videoaulas e uma orelha interativa (recurso *online*) para estudo de localização e aplicação nos principais pontos auriculares. As palestras presenciais seguem um roteiro estruturado de técnicas de palpação auricular, prática de inserção de sementes auriculares e discussão de casos clínicos, sob supervisão de instrutores treinados^(16)^.
O conteúdo incluiu evidências, indicações para dor, contraindicações, aplicação, segurança, mecanismo de ação e como negociar em caso de barreiras de credenciamento. Os *workshops* usaram os métodos de ensino como a aprendizagem baseada em problemas, infoentretenimento, simulação, “ensino de habilidades em quatro estágios de *Peyton*” e “ensino em movimento”. O aprendizado pré-curso foi fornecido principalmente por meio de vídeos sobre: “Visão geral da aplicação, adequação e seleção de casos do ED BFA”; “Evidências atuais que apoiam a acupuntura auricular ED”; “Como realizar o BFA”; “Mecanismos de acupuntura auditiva”; e “Segurança do BFA”. Incluídos no pacote pré-curso estavam vídeos do Dr. Richard Niemtzow sobre “Abertura da embalagem, retirada de aplicador e demonstração do tamanho da agulha” e “Aplicação dos pontos BFA em sequência em uma orelha”, aplicação das fitas BFA e *DuoDERM* e da remoção segura das agulhas no ambiente de ED. As pré-leituras atribuídas incluíram artigos de revisão sistemática. A simulação nos modelos de orelhas de silicone ajudou a ensinar a destreza necessária no uso das agulhas auriculares *aiguille semi-permanente* (*Sedatelec, Irigny*, França)^(18)^.
Curso 1: não especifica. Curso 2: faz parte do curso o atendimento de 24 usuários, com relato dos atendimentos e envio à tutora do curso. Curso 3: o curso conta com uma instrutora. A parte teórica abordou a origem, funcionamento, atuação e finalidade da auriculoterapia. Na prática, foi realizada a aplicação nos colegas do curso^(19)^.
Protocolo de trauma auricular, com cinco agulhas; protocolo de trauma auricular, com seis agulhas; BFA, com agulhas ASP^(20)^.
O treinamento didático compreendeu conteúdos oriundos de livros didáticos, artigos e sites, apresentados em cinco palestras: (1) visão geral e perspectiva teórica da auriculoterapia; (2) terminologia anatômica da orelha externa; (3) pontos mestres auriculares, musculoesqueléticos e sensoriais pontos auriculares; (4) procedimentos de diagnóstico e tratamento auricular; e (5) protocolos de APA comumente usados para dor relacionada ao câncer. Para o treinamento prático, o aprendizado foi reforçado por meio de aprendizado imersivo, demonstração e devolução. Os enfermeiros praticaram a identificação dos pontos auriculares apropriados colocando um alfinete em orelhas de silicone. Em seguida, eles praticaram o uso de um localizador para identificar os pontos auriculares em uma área selecionada da dor relacionada ao câncer e à colocação de sementes da APA em uma enfermeira voluntária enquanto eram observados e orientados pelo professor. Para concluir com êxito o curso prático, os enfermeiros tiveram que demonstrar competência em APA por meio de uma lista de verificação de habilidades, incluindo o uso de um localizador para encontrar os pontos correspondentes à dor no corpo, colocação de sementes e estimulação dos pontos auriculares^(21)^.
Ao decorrer da etapa EaD, que totalizou 75 horas, os cinco módulos davam a dimensão do que é a auriculoterapia, como realizar diagnóstico, possibilidades de abordagens e como poderia ser agregada na rotina de trabalho, trazendo alguns exemplos e instigando a pensar na prática e no processo de trabalho com a auriculoterapia^(22)^.
A primeira etapa é classificada como autoinstrucional, enquanto a segunda é chamada de preceptoria, que possibilita pôr em prática o que foi elaborado teoricamente nos módulos. O primeiro módulo se inicia com o processo histórico da presença das PIC no SUS e uma breve introdução sobre a auriculoterapia. Os três módulos a seguir contemplam três abordagens dessa técnica: reflexologia, MTC e biomedicina. Cada módulo se dedica a uma dessas racionalidades terapêuticas, organizando como é a condução para diagnosticar e definir tratamento. O último módulo se dedica a sistematizar como pode ser realizada a entrada desta prática nos atendimentos do SUS^(23)^.

*APS - Atenção Primária à Saúde; MTC - medicina tradicional chinesa; BFA- Battlefield Acupuncture; NADA - National Acupuncture Detoxification Association; PIC - práticas integrativas e complementares; SUS - Sistema Único de Saúde; EaD - educação a distância; APA - acupressão do ponto auricular; ED – emergency department.*

### Conteúdo programático dos cursos de auriculoterapia e/ou acupuntura auricular

Três estudos recuperados não apresentam descrição do conteúdo programático^(11,13,17)^, enquanto os demais abordam aspectos da formação em auriculoterapia, como base biológica, histórico, prática clínica, protocolos de tratamento e métodos de ensino. Entre os métodos de ensino, destacaram-se ensino baseado em evidências^(14,18)^, simulação e aplicação prática, para garantir as competências necessárias na auriculoterapia, como pode ser visualizado no Quadro 3.

De modo geral, os cursos abrangem elementos teóricos e práticos, com foco em segurança, técnica adequada e identificação correta dos pontos auriculares. A única formação que descreve o ensino na MTC é brasileira^(16)^, pois apresenta uma introdução às explicações médicas e biomédicas da MTC, incluindo neurofisiologia e pesquisa científica. Os autores justificam essa escolha por dois motivos: (1) didático: com base na experiência de ensino presencial, o conteúdo *online* (reflexologia – MTC – biomedicina) seria de fácil compreensão; (2) epistemológico: contextualizando o material em cada uma das três abordagens, evitou-se reduzir ou explicar uma abordagem com base nas outras – geralmente a biomedicina. Assim, o curso utiliza os termos da MTC e da reflexologia auricular em sua abordagem, não aplicando os procedimentos metodológicos típicos utilizados na biomedicina.

De acordo com os conteúdos descritos nos documentos^(12,14,18)^, a formação em auriculoterapia ocorre por meio de um currículo que abrange a base biológica fundamental, aplicações clínicas, ensino teórico e prático, protocolos específicos, e métodos de ensino baseados em evidências. Os programas de treinamento incluem aulas teóricas, aplicações práticas, simulações e discussão de casos clínicos. No que se refere a métodos de ensino, percebe-se que as formações no protocolo BFA fazem o uso de orelhas de silicone^(14,15,18,21)^ para manipular as agulhas antes do treinamento em pessoas. Destacamos estudo^(18)^ no qual são descritos métodos de ensino como a aprendizagem baseada em problemas, infoentretenimento, simulação, “ensino de habilidades em quatro estágios de Peyton” e “ensino em movimento”.

A Escala de Confiança de Grundy (1993)^(24)^ foi aplicada com os enfermeiros oncológicos antes e depois da formação, através de uma medida de autorrelato composta por um item adaptado da escala: de modo geral, quão confiante você está de que é capaz de praticar APA com sucesso? O item foi medido por meio de uma escala Likert de 10 pontos, variando de 0 (nada confiante) a 10 (extremamente confiante). Assim, a pontuação média na medida de confiança na prática de APA aumentou de 0 antes do *workshop* para 6,5 (DP = 0,84, variação de 5 a 10) após o *workshop* ^(21)^.

### Características dos formadores dos cursos de auriculoterapia e/ou acupuntura auricular

Nem todas as produções que compuseram o *corpus* deste escopo detalham quem foram as pessoas que conduziram as formações ou pensaram o conteúdo dos cursos. Assim, estudo^(15)^ contou com um instrutor da BFA foi treinado pelo Dr. Niemtzow no Centro de Acupuntura da Força Aérea. Já no estudo^(16)^, uma equipe multidisciplinar criou o conteúdo do curso de auriculoterapia. A maioria dos membros da equipe tinha experiência anterior ou atual como profissional convencional da APS (três médicos, dois enfermeiros, um farmacêutico e um fisioterapeuta). Todos eram acupunturistas com experiência no uso e/ou ensino de auriculoterapia/acupuntura. Essa equipe foi responsável pela preparação de todo o material do curso e formou o comitê gestor do curso.

Os professores do curso^(18)^ eram profissionais de emergência com qualificações formais em acupuntura geral, médicos de clínica geral (medicina de família e comunidade) do corpo docente de exames do *Australian Medical Acupuncture College* e profissionais de MTC. Os profissionais de emergência compreenderam a combinação de casos e quais os problemas de analgesia que poderiam ser resolvidos. Além disso, eles sabiam como ajustar as técnicas de acupuntura auricular ao ambiente do pronto-socorro, ao mesmo tempo em que compreendiam a mentalidade dos participantes. Os professores de medicina geral (medicina de família e comunidade) e os praticantes de MTC contribuíram na seleção precisa de pontos, localização e técnicas de agulha. O corpo docente da MTC tinha excelente conhecimento sobre as teorias cosmológicas chinesas, mas apresentou ressalvas ao modelo biomédico, o que foi observado no *feedback* formal.

Celia Hildebrand, após uma palestra em 2017 na *Uzhhorod National University College of Medicine* sobre o uso da acupuntura auricular para dores e vícios, recebeu uma bolsa *Fulbright* para desenvolver um currículo de acupuntura auricular na Ucrânia. O treinamento foi organizado pela *Uzhhorod National University College of Medicine*, com duração de 14 horas, e o mesmo resultou em uma certificação e créditos de Educação Médica Continuada. O currículo foi posteriormente melhorado e entregue novamente em agosto de 2022, e novamente revisado e ministrado em maio de 2023. Em junho de 2023, o curso foi ministrado pela primeira vez em Lviv, Ucrânia^(20)^.

## DISCUSSÃO

Algumas MAC são utilizadas há milhares de anos. Sendo assim, cabe a reflexão de que o registro e a documentação quanto ao uso das mesmas podem estar aumentando no mundo. Além disso, instituições preocupadas com a formação, certificação e informações também crescem. Por exemplo, tem-se nos EUA o *National Center for Complementary and Alternative Medicine*, que realiza e apoia pesquisas, e fornece informações sobre produtos e práticas de saúde complementares^(25)^. No que tange à formação, os países são incentivados a avaliar e reconhecer os documentos de referência da OMS (*benchmarks*) como padrões mínimos, conforme sejam relevantes para o seu contexto nacional^(7)^.

Há 12 anos, em outubro de 2012, aconteceu o *First International Congress for Educators in Complementary and Integrative Medicine*, na capital dos EUA, com objetivo de avançar no campo da educação em medicina complementar e integrativa através do compartilhamento de melhores práticas no desenvolvimento, implementação, avaliação e disseminação de currículos e métodos de ensino^(26)^. O congresso pode ser apontado como um marco no que tange à formação nessas práticas.

A Estratégia de Medicina Tradicional da OMS 2013-2023 (estendida até 2025) é um conjunto de diretrizes que visa apoiar os estados-membros na implementação de políticas e planos de ação que fortaleçam a importância da medicina tradicional. Entre os desafios citados na mesma, estão a formação e treinamento de praticantes de medicina tradicional^(2)^.

Voltando a atenção para o ensino e formação da auriculoterapia, formações *online* vêm sendo desenvolvidas, como o curso do Dr. Terry Oleson, especialista e líder mundial em auriculoterapia, que fornece certificação pelo *Auriculotherapy Certification Institute* (ACI)^(27)^. O ACI é um instituto criado em 1999 para oferecer treinamento e certificação em auriculoterapia nos EUA, não isentando os praticantes de cumprir as regulamentações estaduais. O ACI apresenta três tipos de certificação em auriculoterapia: auriculoterapia por estimulação transcutânea; acupuntura auricular com inserção de agulha; e reflexologia auricular com manipulação tátil^(27)^. Ainda, outro curso de relevância internacional é do Dr. Raphaël Nogier, médico e pesquisador^(28)^.

A existência de cursos de renome e poucas experiências de ensino de auriculoterapia e/ou acupuntura auricular registradas na literatura^(12)^ acaba por direcionar a discussão para exemplos da acupuntura, prática que parece possuir padrões de formação mais definidos. Assim, a fim de problematizar algumas questões que devem ser levadas em consideração nas formações de auriculoterapia, de acordo com a literatura mundial, algumas lacunas e possibilidades serão apontadas.

Na Austrália, desde julho de 2012, foi implantado um esquema nacional de registro e credenciamento para profissões de saúde que incorpora o registro de acupunturistas no Conselho de Medicina Chinesa da Austrália. Revisão bibliográfica identificou estudos sobre padrões de educação em acupuntura, bem como pesquisa de órgãos de definição de padrões e provedores de cursos no país, porém concluiu que há pouca literatura sobre os padrões de educação em acupuntura na Austrália, apesar de sua prática por uma ampla gama de profissionais de saúde^(29)^. Ainda, no país, os profissionais de saúde australianos que atuam com medicina complementar são supervisionados pela *Australian Health Practitioner Regulation Agency* desde 2010, incluindo acupunturistas. Pesquisa conclui que as qualificações dos profissionais, os padrões educacionais e o escopo dos procedimentos relacionados à medicina complementar são gerenciados por meio de regulamentações legais dos governos federal e estadual^(30)^. Não foram encontradas evidências sobre padrões ou registros para prática da auriculoterapia de forma específica no país.

Estudo brasileiro^(19)^ conclui que os processos formativos em práticas integrativas e complementares em saúde (PICS) (nomenclatura das MTCI ou MAC no Brasil) são heterogêneos, deficientes e limitados. Ainda, demonstram a carência de ordenação de formação de recursos humanos e desenvolvimento de estratégias de qualificação nas PICS. Estudo realizado em Florianópolis, Santa Catarina, Brasil, apresenta um dado relevante no que tange à inexistência do emprego de recursos diagnósticos e de anamnese desses saberes tradicionais da MTC antes da aplicação da auriculoterapia. Pesquisador observou que em tais práticas é realizado o diagnóstico seguindo a lógica biomédica padrão e, posteriormente, a intervenção em PICS^(13)^. Assim, tal aspecto reforça a necessidade de uma diretriz direcionando e qualificando a formação em auriculoterapia de acordo com as bases que originaram a prática. Dado corrobora o conteúdo curricular identificado na presente revisão, em que apenas os cursos da UFSC relataram o estudo da MTC.

Tendo em vista as contribuições que as PICS (entre elas a auriculoterapia) podem ter na integralidade do cuidado em saúde, em especial experiências positivas da APS brasileira, estudo de revisão refere que “[...] há escasso investimento no seu ensino, sendo pouco dominada pelos alunos e professores da graduação dos cursos da área da saúde. Dessa forma, urge que as PICs sejam consideradas para a sua inclusão na matriz curricular do ensino em saúde [...]”^(31)^.

Estudo realizado no Brasil, com 24 gerentes de Unidades Básicas de Saúde na Região Metropolitana de Goiânia, Goiás, sendo a maioria enfermeiros, refere a falta da formação em serviço dos profissionais como uma barreira educacional para implementação das PICS na APS. Os gerentes afirmam a falta de educação continuada sobre PICS como oferta pública, cabendo aos profissionais a busca pela formação. Ademais, citam o curso presencial de auriculoterapia ofertado pela Secretaria de Saúde de Goiânia de forma gratuita. A formação em PICS é um dos pontos críticos para a ampliação efetiva dessas práticas^(32)^.

A importante iniciativa do *Defense Veteran’s Center for Integrative Pain Management* é a educação e formação de prestadores multidisciplinares em estratégias integrativas de gestão da dor, evidenciando a importância da formação, bem como o financiamento para subsidiar a mesma. O mesmo artigo desenvolveu um currículo de acupuntura auricular para treinar mais de 2.700 profissionais do *The Department of Defense and Veterans Affairs* com responsabilidades no tratamento da dor desde 2016^(14)^. Para esse tratamento da dor, vários autores utilizam a BFA, desenvolvida em 2001, por Richard C. Niemtzow, oncologista de radiação da Força Aérea dos EUA, que adaptou a técnica de acupuntura auricular para ser usada como método de alívio rápido da dor no campo de batalha. Trata-se de uma técnica que utiliza agulhas semipermanentes que são colocadas em pontos de acupuntura auriculares e que podem permanecer na orelha por três a quatro dias^(33)^.

O BFA, embora tenha suas bases na milenar MTC, pode ser considerada uma modalidade nova no meio de uma comunidade médica de base farmacêutica e intervencionista^(34)^. Esta modalidade oferece um novo paradigma, com uma habilidade prática que está fora das influências atuais da indústria e do setor farmacêutico. Os autores concluem que a mesma pode ser facilmente incorporada em um currículo de residência médica, especialmente aqueles com treinamento que gira em torno da qualidade de vida, controle da dor e função dos pacientes^(15)^.

Quando fala-se em BFA para alívio da dor em atendimentos de pronto-socorro, enfermeiros mostram-se preocupados com a prescrição excessiva de opiáceos, e entendem serem a categoria profissional ideal para aprender auriculoterapia para uso nesses casos, devido à proximidade com o paciente e por suas habilidades para utilização da BFA, que pode ser aplicada na triagem^(18)^.

### Limitações do estudo

Ao passo que as principais diretrizes de método e relatório para revisão de escopo foram seguidas, a dificuldade de acesso a algumas produções para análise na íntegra, resultando em exclusão, configura-se uma limitação. Ainda, a não padronização de informações das produções sobre a formação em auriculoterapia/acupuntura auricular dificultou outras comparações e inferências.

### Contribuições para as áreas da saúde, enfermagem, ou políticas públicas

As evidências apontam para uma carência de pesquisas que orientem, regulamentem ou direcionem minimamente a formação de profissionais da saúde em auriculoterapia/acupuntura auricular. Dessa forma, trazendo para a discussão outras práticas com formações mais delimitadas, como a acupuntura, as evidências científicas deste escopo contribuem ao direcionar e proporcionar subsídios para a construção de padrões mínimos de formação, de acordo com cada realidade e profissão. Logo, ao trazer características da formação de profissionais da saúde em auriculoterapia/acupuntura auricular em nível mundial, esta revisão poderá servir de base para elaborar, qualificar, aprofundar, avaliar e monitorar diretrizes para formação de profissionais da saúde em auriculoterapia/acupuntura auricular.

## CONSIDERAÇÕES FINAIS

Foi possível mapear as características da formação de profissionais da saúde em auriculoterapia/acupuntura auricular em nível mundial. Em suma, verificou-se que a formação em auriculoterapia ocorre de forma distinta no mundo. As convergências são entre Brasil e França, que formam auriculoterapeutas. Em demais países, como EUA, Austrália e Ucrânia, as formações são direcionadas para uso e aplicação de protocolos específicos, como BFA e NADA. Somando a isso, a modalidade semipresencial foi observada apenas nas formações no Brasil e Austrália. Nenhum curso descrito afirmou ser totalmente *online*, e a carga horária variou de 228 até 3 horas de ensino. A única formação que descreve o ensino na MTC é brasileira. No que tange aos formadores, dois estudos tiveram uma equipe multiprofissional elaborando e ministrando o conteúdo da formação, sendo um deles no Brasil e outro na Austrália.

Não foram recuperadas investigações que avaliaram diretrizes de formação em auriculoterapia/acupuntura auricular. Sendo assim, sugerem-se pesquisas futuras nessa linha, para o avanço na oferta de cuidados para a população a partir de uma formação qualificada, contemplando um dos desafios da Estratégia de Medicina Tradicional da OMS 2013-2023 (estendida até 2025). Ainda, é preciso avançar em pesquisas que elaborem referências para estabelecer sistemas de treinamento, exame e licenciamento que apoiam a prática qualificada da auriculoterapia/acupuntura auricular no mundo, respeitando cada contexto e profissão, a fim de chegar em parâmetros mínimos e legislação específica de formação/ensino de auriculoterapia/acupuntura auricular.

## Data Availability

Os dados de pesquisa estão disponíveis em repositório: https://osf.io/5j86r/.
